# Altered Dopamine Signaling in Extinction-Deficient Mice

**DOI:** 10.1523/ENEURO.0174-25.2025

**Published:** 2025-11-18

**Authors:** Ozge Gunduz-Cinar, Eva Maria Fritz, Maya Xia, Elise Van Leer, Nevin Crow, Andrew Holmes, Nicolas Singewald

**Affiliations:** ^1^Laboratory of Behavioral and Genomic Neuroscience, National Institute on Alcohol Abuse and Alcoholism, National Institutes of Health, Bethesda, Maryland 20852; ^2^Department of Pharmacology and Toxicology, University of Innsbruck, Innsbruck 6020, Austria

**Keywords:** DAT-Cre, dopamine, fear extinction, prediction error

## Abstract

A central mechanism of exposure-based cognitive behavioral therapy for anxiety and trauma-related disorders is fear extinction. However, the mechanisms underlying fear extinction are deficient in some individuals, leading to treatment resistance. Recent animal studies demonstrate that upon omission of the aversive, unconditioned stimulus (US) during fear extinction, dopamine (DA) neurons in the ventral tegmental area (VTA) produce a prediction error (PE)-like signal. However, whether this VTA-DA neuronal PE-like signal is altered in animals exhibiting deficient fear extinction has not been studied. Here, we used a mouse model of impaired fear extinction [129S1/SvImJ (S1) inbred mouse strain] to monitor and manipulate VTA-DA neurons during extinction. Male DAT-Cre mice backcrossed onto an S1 background (S1-DAT-Cre) exhibited impaired extinction but normal VTA-DA neuron number, as compared with BL6-DAT-Cre mice. In vivo fiber photometry showed that impaired extinction in male S1-DAT-Cre mice was associated with abnormally sustained US omission-related VTA-DA neuronal calcium activity during extinction training and retrieval. Neither in vivo optogenetic photoexcitation of VTA-DA neuronal cell bodies nor their axons in the infralimbic cortex was sufficient to rescue deficient extinction in male S1-DAT-Cre mice, at least within the optogenetic and behavioral parameters used. These data suggest that alterations in the activity of VTA-DA neurons during extinction learning and retrieval may be associated with deficient fear extinction in male S1 mice and could potentially contribute to extinction impairments in patient populations.

## Significance Statement

This study investigated VTA-DA neuronal function in mice exhibiting deficient fear extinction.

## Introduction

Dopamine (DA) is a catecholaminergic neurotransmitter mediating functions including motor control, motivation, cognitive functioning, and various neuropsychiatric disease states characterized by deficits in these functions. Prior studies have linked polymorphisms in genes for DA transporter (DAT), DA receptor 4 (DRD4), DA receptor 3 (DRD3), and catechol-O-methyltransferase, with heightened risk for post-traumatic stress disorder (PTSD), greater PTSD symptom severity, impaired cognitive function, and impaired fear extinction in PTSD patients (Dragan and Oniszczenko, 2009; Drury et al., 2009; Kolassa et al., 2010; Norrholm et al., 2013; Wolf et al., 2014; Deslauriers et al., 2018; Havelka Mestrovic et al., 2018). There are also reports of increased urinary excretion of DA and other catecholamines (Yehuda et al., 1992; Lemieux and Coe, 1995; Spivak et al., 1999; Glover et al., 2003). In the brain, an elevated DAT density in the striatum of PTSD patients has been reported, possibly reflecting lower synaptic DA availability or, alternatively, a compensatory response to higher DA turnover (Hoexter et al., 2012).

The role of DA in fear extinction is further suggested by pharmacological studies evaluating the effect of DA receptor agonists or drugs augmenting extracellular DA levels on exposure-based attenuation of fear memories (Abraham et al., 2014; Singewald et al., 2015). For example, increasing DA signaling via systemic administration of the DA reuptake inhibitor methylphenidate was found to facilitate extinction memory consolidation in the C57BL/6 (BL6) and BALB/c inbred mouse strains (Abraham et al., 2012; Jager et al., 2019). Moreover, postextinction administration of a single dose of the DA precursor l-DOPA enhanced extinction memory retrieval and prevented the return of fear in studies in extinction-competent BL6 mice and humans (Haaker et al., 2013; Gerlicher et al., 2018; Andres et al., 2024). Furthermore, the extinction-impaired phenotype of the 129/SvImJ (S1) inbred mouse strain (Whittle et al., 2010; Fitzgerald et al., 2014; Park and Chung, 2020) was rescued by l-DOPA administration, although this effect was not long-lasting (Whittle et al., 2016; Sartori et al., 2024).

Despite these findings, studies investigating DA-targeted treatments in PTSD and anxiety disorders remain scarce. One case-report study reported that DA-modulating psychostimulants may attenuate PTSD symptoms (Houlihan, 2011). Another, conducted in female PTSD patients, found that l-DOPA administration during postextinction consolidation enhanced extinction-related activity in the amygdala during resting-state scans, as compared with placebo-treated controls, but did not find evidence of improved extinction recall the next day (Cisler et al., 2020). However, l-DOPA–treated individuals exhibited a reduced skin conductance response during fear reinstatement, suggesting the role of DA in the formation of persistent extinction memories that protects against fear reinstatement in PTSD (Cisler et al., 2020).

The majority of DAergic cell bodies are located in two regions of the midbrain: the substantia nigra and ventral tegmental area (VTA; Bjorklund and Dunnett, 2007). VTA-DA neurons projecting to limbic and cortical areas have been linked to the modulation of various positive and negatively valenced behaviors, including fear extinction (Bukalo et al., 2014; Hariri and Holmes, 2015; Likhtik and Johansen, 2019; Salinas-Hernandez and Duvarci, 2021; Singewald et al., 2023; Duvarci, 2024; Hamati et al., 2024). Prediction error (PE; Rescorla and Wagner, 1972) is a DAergic signal encoding the discrepancy between expected and actual outcomes (Schultz, 2016). Accordingly, the unexpected omission of the aversive unconditioned stimulus (US) during extinction training generates a PE-like signal that manifests as a change in the activity of a subset of mouse and rat VTA-DA neurons (Luo et al., 2018; Salinas-Hernandez et al., 2018; Cai et al., 2020; Salinas-Hernandez and Duvarci, 2021). VTA-DA neuronal activity in extinction-competent BL6–background mice increases at the time of US omission during early extinction training and this response decreases with extinction (Salinas-Hernandez et al., 2018, 2023). Such extinction-related PE–like signaling in BL6-background mice is primarily evident in medial VTA-DAergic neurons, whereas lateral VTA neuronal activity changes at CS onset (Cai et al., 2020; Salinas-Hernandez et al., 2023). The functional importance of these neuronal correlates is evidenced by the finding that optogenetic silencing of medial VTA-DA neurons during US omission is sufficient to impair extinction in BL6-background mice (Cai et al., 2020).

These previous findings demonstrate that VTA-DA neurons generate a putative PE-like signal that underlies fear extinction and implies that abnormalities in this mechanism may contribute to impairments in fear extinction. In turn, these preclinical findings suggest rectifying VTA-DA neuronal dysfunction as a novel therapeutic approach to facilitate extinction. To date, however, there has been limited investigation of VTA-DA neuronal function in the context of deficient fear extinction. To address this important gap in the literature in the current study, we used in vivo fiber photometry and optogenetics to respectively monitor and manipulate VTA-DA neurons during fear extinction in a mouse model (S1 inbred strain) previously shown to exhibit impaired extinction (Camp et al., 2009; Whittle et al., 2010, 2013; Singewald and Holmes, 2019).

## Materials and Methods

### Animals and husbandry

All experiments were carried out in compliance with national and international guidelines for animal welfare and appropriate guides for Care and Use of Laboratory Animals and approved by appropriate Animal Care and Use Committees. Male 129S1/SvImJ (S1) mice, C57BL/6J (BL6) mice, as well as DAT-Cre mice [B6.SJL-Slc6a3tm1.1(cre)Bkmn/J; JAX Stock No. 006660] on a C57BL/6J background (BL6-DAT-Cre) or backcrossed to a 129S1/SvImJ background (S1-DAT-Cre) for at least nine generations (11–16 weeks at the start of experiments or surgery) were bred in-house or purchased from JAX (The Jackson Laboratory) and habituated in the animal facility for at least 1 week. Animals were housed at constant temperature (22 ± 2°C) and humidity (40–60%) under a 12 h light/dark cycle (lights on/off at 07:00/19:00) with *ad libitum* access to food and water. All mice were held in groups (maximum of five per cage) and single-housed following surgery. Exact *n* numbers for each experiment are given in the corresponding figure legends.

### Surgical procedures for fiber photometry

Surgical procedures were performed under isoflurane anesthesia (Forane, USP, Baxter) provided at a constant rate (2.0–5.0% isoflurane, oxygen flow rate 1 L/min; Anesthesia System, E-Z Systems), while the animal was head-fixed in a stereotaxic frame (Kopf Model 942, David Kopf Instruments). The animal was placed on a heating pad, and the breathing rate was constantly monitored. The eyes of the animal were covered with eye lubricant ointment (LubriFresh PM, Major Pharmaceuticals) to prevent desiccation. As analgesic, ketoprofen was subcutaneously injected (5 mg/kg).

Using a dental precision driller, one craniotomy was made at the following coordinates (from the bregma): AP −3.20 mm and ML ±0.40 mm. An AAV construct (Addgene) for the Cre-dependent expression of GCaMP6f (AAV5-pAAV-CAG-FLEX-GCaMP6f-WPRE-SV40, Addgene_100835; 1.3 × 1,013 vp/ml) was delivered unilaterally (0.2 µl) into the VTA at DV −4.6 mm. This was done via a 0.5 µl microliter syringe equipped with a 32 G needle (Neuros 7000.5, Hamilton) and at a rate of 20 nl/min [UMP3 with Micro4 Controller, World Precision Instruments (WPI)]. After the injection, the needle was left in place for another 5–10 min to ensure diffusion. Then, a single fiber (0.66 NA, ø 400 μm, 6 mm length; MFC_400/430-0.66_6mm_SM3_FLT, Doric Lenses) was implanted unilaterally directly above the VTA (coordinates relative to the bregma: AP −3.20, ML 0.40, DV −4.6, 0° angle). Two additional craniotomies were made in which jeweler's screws were mounted to build a fundament using dental cement (OrthoJet, Lang Dental Manufacturing) and ensure fixation of the implants to the skull. To reduce postoperative pain, ketoprofen was administered subcutaneously directly after surgery and for the following 3 d (5 mg/kg). After surgery, recovery of the animals and AAV expression were allowed for 4–5 weeks, before the start of behavioral experiments.

### Surgical procedures for optogenetics

Surgical procedures were performed under isoflurane anesthesia (Vetflurane, Virbac) provided at a constant rate (1.9–2.5% isoflurane, air flow rate 190–220 ml/min; Univentor 410 Anesthesia Unit, AgnThos), while the animal was head-fixed in a stereotaxic frame (Kopf Model 962, David Kopf Instruments). The animal was placed on a heating pad, and the breathing rate was constantly monitored. The eyes of the animal were covered with eye cream (Bepanthen Augen- und Nasensalbe, Bayer) to prevent desiccation. As an analgesic, meloxicam was subcutaneously injected (0.5 mg/kg; Metacam, B. Braun Melsungen). A 1% lidocaine solution (Xylocaine 2% Ampullen, Aspen Pharmacare) was locally applied to the scalp before a rostrocaudal incision was made.

Using a dental precision driller, two (VTA fiber implantations) or four [infralimbic cortex (IL) fiber implantations] craniotomies were made at the following coordinates (from the bregma): AP −3.20 mm, ML ±0.5 mm (VTA) and AP +1.40 mm, ML ±1.3 mm (IL). AAV constructs (UNC Vector Core) for the Cre-dependent expression of channelrhodopsin 2 (ChR2) and mCherry (AAV5-EF1a-DIO-ChR2(H134R)-mCherry, Addgene_20297; 3.4 × 1,012 vp/ml) and mCherry only (AAV5-EF1a-DIO-mCherry, Addgene_114471; 5.4 × 1,012 vp/ml) were delivered bilaterally (0.35 µl/hemisphere) into the VTA at DV −4.6 mm. This was done via a 0.5 µl microliter syringe equipped with a 32 G needle (Neuros 7000.5, Hamilton) and at a rate of 35 nl/min (UMP3 with Micro4 Controller, WPI).

After the injection, the needle was left in place for another 5–10 min to ensure diffusion. Then, single (IL, 0.39 NA, ø 200 μm, cleaved to 4 mm length; CFMLC12U-20, Thorlabs) or dual fibers (VTA, 0.22 NA, ø 200 μm, 8.5 mm length; DFC_200/240-0.22_8.5mm_GS1.0_FLT, Doric Lenses) were implanted bilaterally above IL (DV −1.90 mm, 20° angle) or VTA (DV −4.10 mm, 0° angle). Two additional craniotomies were made in which jeweler's screws were mounted to build a fundament using dental cement (Paladur, Heraeus-Kulzer) and ensure fixation of the implants to the skull. To reduce postoperative pain, a single dose of buprenorphine was administered subcutaneously directly after surgery (0.5 mg/ml; Bupaq, Richter Pharma) and meloxicam was provided via drinking water (1 mg/ml; Metacam, B. Braun Melsungen) for the following 3 d. After surgery, recovery of the animals and AAV expression were allowed for 4–5 weeks, before beginning behavioral experiments.

### Behavioral testing for S1-DAT-Cre phenotyping

Before the start of behavioral experiments, all animals were habituated to the experimenter and the handling procedure for at least 3 d. Fear conditioning (FC) and extinction procedures were conducted based on previously employed methods (Gunduz-Cinar et al., 2019) and here controlled using ANY-maze behavioral tracking software (Stoelting Europe) with a compatible FC system (Ugo Basile). All sessions were digitally video recorded for later analysis.

FC (Context A): The conditioning chamber was a 17 × 17 × 25 cm cubicle with transparent walls, a Lucite lid, and a metal rod floor that was cleaned with water and illuminated with white light to 600 lux. The room in which FC was performed was brightly illuminated with white light. After a 120 s acclimation period, three pairings of a CS (10 kHz sine tone, 75 dB, 30 s) with a coterminating US (scrambled footshock, 0.6 mA, 2 s) were delivered with a variable (30–120 s) intertone interval. After the final pairing, a 120 s no-stimulus consolidation period followed, before the mice were returned to their home cage.

Fear extinction training (EXT; Context B): The extinction context was a 17 × 17 × 25 cm cubicle with black and white checked walls, an open top and a solid gray floor that was cleaned with 1% acetic acid and illuminated with red light to 5 lux. The room in which fear extinction training was performed was dimly illuminated with white light. After a 120 s acclimation period, 20 CS presentations with a variable (5–30 s) intertone interval were delivered. After the final presentation, a 120 s no-stimulus consolidation period followed, before the mice were returned to their home cage.

Extinction retrieval (RET; Context B): For extinction retrieval, the mice were again placed in the extinction context (Context B) 24 h following extinction training. After a 120 s acclimation period, 5 CS presentations with a variable (5–30 s) intertone interval were delivered. After the final presentation, a 120 s no-stimulus consolidation period followed, before the mice were returned to their home cage.

For quantification of freezing behavior, the time spent freezing was manually scored and quantified using a custom-written MATLAB script (MathWorks). All freezing values are presented as the percentage of the analyzed time period or trial blocks (e.g., pre-CS, CS, five CS trial blocks).

### Behavioral testing for optogenetics and fiber photometry

Before the start of behavioral experiments, all animals were habituated to the experimenter and the handling procedure for at least 3 d. For optogenetic manipulation and fiber photometry measurements, the animals were connected to the fiber cables via their implants and habituated for 5 (optogenetics) or 10–20 min (fiber photometry) in their home cage before being placed in the corresponding context for behavioral testing.

FC procedures were controlled using ANY-maze behavioral tracking software (Stoelting Europe) with a compatible FC system (Ugo Basile; optogenetics) or Med Associates software and FC system (Med Associates; fiber photometry). Extinction procedures were controlled using ANY-maze behavioral tracking software (Stoelting Europe) and corresponding AMi-2 audio and optogenetics interfaces (optogenetics) or Ethovision software (Noldus) with compatible hardware (fiber photometry). All sessions were digitally video recorded for later analysis.

FC (Context A): The conditioning chamber was a 26 × 26 × 35 cm (optogenetics) or a 30 × 25 × 25 cm (fiber photometry) cubicle with transparent walls, an open top and a metal rod floor that was cleaned with water and illuminated with white light to 600 lux. The room in which FC was performed was brightly illuminated with white light. After a 180 s acclimation period, five parings of a CS (10 kHz sine tone, 75 dB, 28 s) followed by an US (scrambled footshock, 0.6 mA, 2 s) were delivered with a 90 s intertone interval. After the final pairing, a 120 s no-stimulus consolidation period followed, before the mice were returned to their home cage.

Fear extinction training (EXT; Context B): The extinction context was a 24 × 32 × 30 cm cubicle with white walls, an open top, and a solid yellow floor (optogenetics) or a transparent 27 × 27 × 14 cm container with some bedding material covering the floor (fiber photometry) that was cleaned with 1% acetic acid and illuminated with red light to 5 lux. The room in which fear extinction training was performed was dimly illuminated with red light. After a 180 s acclimation period, 25 CS presentations with a 7 s (optogenetics) or 20 s (fiber photometry) intertone interval were delivered. After the final presentation, a 120 s no-stimulus consolidation period followed, before the mice were returned to their home cage.

Extinction retrieval (RET; Context B): For extinction retrieval, the mice were again placed in the extinction context (context B) 24 h following extinction training. After a 180 s acclimation period, five CS presentations with a 7 s (optogenetics) or 20 s (fiber photometry) intertone interval were delivered. After the final presentation, a 120 s no-stimulus consolidation period followed, before the mice were returned to their home cage.

For quantification of freezing behavior, the time spent freezing was either manually scored and quantified using a custom-written MATLAB script (MathWorks) or measured automatically using ANY-maze behavioral tracking software (Stoelting Europe) according to predefined thresholds that were validated by manual scoring. All freezing values are presented as the percentage of the analyzed time period or trial blocks (e.g., pre-CS, CS, five CS trial blocks).

### Fiber photometry

For recording of fluorescent signals during all behavioral tests, the implanted optical fibers were connected to mono fiber-optic patch chords with low autofluorescence (0.57 NA, ø 400 µm, 0.60 m length; Doric Lenses). The patch cords were attached to a fiber-optic rotary joint (FRJ_1 × 1_PT_FCM-FCM, Doric Lenses) that was connected to the photometry system. The photometry system consisted of a fluorescence mini cube [FMC6_E1(400–410)_F1(420–450)_E2(460–490)_F2(500–540)_O(570–650)_S, Doric Lenses] that was connected to photoreceivers (NPM_2151_FOA_FC, Doric Lenses) and 465 and 405 nm LED lights (M470F3 and M405F1, Thorlabs). A real-time signal processor was used to sinusoidally modulate LED outputs and collect fluorescent signals at a sampling rate of 1,017 Hz (RZ5P with Synapse Software, Tucker-Davis Technologies).

All data analysis was performed using MATLAB scripts (MathWorks) with minor modifications as previously described (Gunduz-Cinar et al., 2023). Recordings obtained from the 405 nm channel were used to control for autofluorescence, bleaching, and motion artifacts. For this, the 405 nm signal was first aligned to the 465 nm signal by scaling and linear fitting. The change in fluorescence (dF) for each time point (*t*) was calculated by subtracting the fitted control from the raw 465 nm channel signal (Δ*F* = 465 nm signal − fitted 405 nm signal). *Z*-Scores of each time point [*Z*(*t*)] were calculated by normalizing to a predefined baseline period (which was taken 5 s before the first CS onset, otherwise subtracting the average stated in figure legends) by dF at all time points during baseline period from the dF at each time point and dividing to the standard deviation (SD) of all baseline dF:

$$Z\;\mathrm{score}\;\;\mathrm{at}\;\mathrm{time}\;\mathrm{point}\;t=\;Z(t)=\frac{\mathrm{dF}\;(t)-\mathrm{mean}(\mathrm{dF}(\mathrm{baseline}\;\mathrm{period}))}{\mathrm{SD}\;(\mathrm{dF}\;(\mathrm{baseline}\;\mathrm{period}))}.$$

Time-normalized area under the curve (AUC) values were calculated using MATLAB's built-in “trapz” function, which uses trapezoidal numerical integration to calculate the AUC (*Z*-score) between inputted *x*-values (time) on a graph of *z*-score versus time. Time-normalized AUC values for the US (2 s post-US) during conditioning, for the CS onset and CS offset (5 s post CS onset or offset) during conditioning extinction and retrieval were compared with each event's respective (5 s) pre-event baseline. Data analyses were performed using MATLAB R2023b (MathWorks), and graphs were prepared by Graph Pad Prism10.

### Optogenetic manipulations

For optogenetic manipulations during fear extinction, the implanted optical fibers were connected to mono fiber-optic patch chords (0.22 NA, ø 200 µm, 0.75 m length; Doric Lenses). The patch cords were attached to a light-splitting fiber–optic rotary joint (FRJ_1 × 2i_FC-2FC, Doric Lenses) that was connected to a 473 nm laser (MBL-F-473 (150 mW), CNI Optoelectronics Technology). Laser power was adjusted before each animal to 8–10 mW at the fiber tip (PM100D power meter with S120C sensor, Thorlabs). To phasically activate DA neurons on VTA-IL terminals at the time of US omission, 5 ms light pulses were delivered at 20 Hz (5% duty cycle) for 2 s after the offset of the CS. For VTA and VTA-IL activation experiments, the laser was controlled using the ANY-maze behavioral tracking software (Stoelting Europe) with a corresponding AMi-2 optogenetic interface.

### Immunohistochemistry

Tissue extraction and sectioning: After the behavioral experiments involving fiber photometry or optogenetic manipulations, all animals were transcardially perfused with ice-cold 0.9% saline (5 min) and PFA solution (4% in 0.1 M PB; 8 min), pH 7.4, at a flow rate of 5 ml/min (Peri-Star Pro Peristaltic Pump, WPI). The brains were extracted, placed in PFA solution for 24 h for postfixation and then transferred into PB (0.1 M), pH 7.4, until sectioning. All brains were sectioned on a vibrating microtome (VT1000S, Leica Biosystems) at a thickness of 40 µm, and the free-floating sections were stored in PB until further processing.

For mCherry immunostaining, all sections were first washed in a single washing step with phosphate-buffered saline (PBS; 0.05 M), pH 7.4, for 10 min. Then, the sections were incubated with blocking buffer (10% normal goat serum and 1% BSA in 0.05 M PBS with 0.3% Triton X-100), pH 7.4, for 2 h. Next, the primary antibody targeting mCherry was added (polyclonal rabbit, 632496, Takara Bio; 1:500 in blocking buffer diluted 1:10 in 0.05 M PBS, with 0.3% Triton X-100), pH 7.4, overnight at 4°C. On the next day, the primary antibody solution was removed, and the sections were washed with PBS (three times for 10 min) and incubated with the secondary antibody (Alexa Fluor 555, goat anti-rabbit, A-21428, Invitrogen/Thermo Fisher Scientific; 1:500 in blocking buffer diluted 1:10 in 0.05 M PBS, with 0.3% Triton X-100), pH 7.4, for 2 h at room temperature. After removal of the secondary antibody solution, again a washing step with PBS followed (two times for 10 min). Thereafter, the sections were washed once with PB and then mounted onto glass slides. After the slides were dry, they were immediately coverslipped with a DAPI-containing mounting medium (ProLong Gold Antifade Mountant with DAPI, P-36931, Invitrogen/Thermo Fisher Scientific), dried at room temperature overnight and then stored at 4°C.

GCaMP6f expression was sufficiently visible without immunostaining; therefore sections were mounted onto glass slides in PB and counterstained with Hoechst 33342 (5 µg/ml; H1399, Invitrogen/Thermo Fisher Scientific). After the slides were dry, they were immediately coverslipped with a glycerol-based aqueous mounting medium, sealed with nail polish and then stored at 4°C.

For microscopy and histological verification of fiber placements, all sections of interest were 2D-scanned (Stereo Investigator, MBF Bioscience) at 4× magnification in DAPI and CY3 channels using a fluorescent microscope (BX-40, Olympus) with a motorized stage (MAC6000 System, Ludl Electronic Products) or imaged at 4× magnification in DAPI and CY2 channels on a fluorescent microscope with manual stage and cellSens software (BX-41, Olympus). AAV expression and fiber placements were verified according to distinct anatomical landmarks corresponding to the Paxinos stereotaxic mouse brain atlas (Paxinos and Franklin, 2019). Only mice with sufficient AAV expression and correct fiber placements were included in the analysis.

### Immunohistochemistry and stereological analysis of VTA-DA neurons

Tissue extraction and sectioning was performed as described above for the verification of fiber placements.

For immunostaining of tyrosine hydroxylase positive (TH+) neurons, all sections were placed in Tris-buffered saline (TBS; 0.05 M), pH 7.4, and incubated with H_2_O_2_ at a concentration of 0.03% for 30 min. After washing with TBS (three times for 10 min), the sections were incubated with blocking buffer (20% normal goat serum in 0.05 M TBS, with 0.3% Triton X-100), pH 7.4, for 1.5 h. Next, the primary antibody targeting TH was added (polyclonal rabbit, AB152, Merck Millipore; 1:4,000 in 0.05 M TBS, with 2% normal goat serum and 0.3% Triton X-100), pH 7.4, at 4°C for ∼72 h. Again, after three washing steps in TBS (three times for 10 min), the secondary antibody (biotinylated goat anti-rabbit, BA-1000, Vector Laboratories; 1:500 in 0.05 M TBS, with 2% normal goat serum and 0.3% Triton X-100), pH 7.4, at 4°C overnight. After washing in TBS (three times for 10 min), sections were incubated with an avidin–biotin–peroxidase complex solution (Vectastain ABC Kit, Vector Laboratories; in 0.05 M TBS, with 2% normal goat serum), pH 7.4, for 1 h at room temperature. Following three washing steps in Tris-borate buffer (TB; 0.05 M), pH 7.4, the sections were preincubated in 3,3′diaminobenzidine tetrahydrochloride solution (0.5 mg/ml in 0.05 M TB), pH 7.4, for 10 min at RT. The immunoreaction was started by the addition of 3% H_2_O_2_ to a final concentration of 0.003%. The reaction was stopped after 2 min by washing with TBS (three times for 10 min). Finally, the sections were mounted onto gelatine-coated glass slides, air-dried, and dehydrated in ethanol (5 min in each 50, 70, 95, 100, 100%) and xylol (two times for 10 min) before they were immediately coverslipped with Eukitt (Millipore Sigma).

TH+ cell bodies in the VTA were quantified in one per three coronal brain sections of S1 and BL6 mice between bregma −2.92 and −3.80 mm (according to Paxinos and Franklin, 2019). This was done via unbiased stereological analysis (optical fractionator method) on a microscope (BX-40, Olympus) with a motorized stage (MAC6000 System, Ludl Electronic Products) and a computer-assisted image analysis system (Stereo Investigator Software, MBF Biosciences). The following counting parameters and settings were used: counting frame 50 × 50 µm; grid size 150 × 80 µm; section evaluation interval 3; and magnification 2× and 100× (oil). TH+ cell bodies are reported per hemisphere. The Gundersen coefficient errors (*m* = 1) were ≤0.4.

### Statistical analysis

The GraphPad Prism 9 and 10 software (GraphPad Software) or MATLAB (MathWorks) were used for generating graphs and performing statistical analysis. Results from all statistical analysis are summarized in a table as Extended Data Figure 1-1. All datasets were tested for normality using a Shapiro–Wilk test or a D’Agostino and Pearson’s test. Two-tailed, unpaired Student's *t* test (two-group comparisons) and one-way ANOVA with a Šídák post hoc test (multigroup comparisons) was used for normally distributed data without or with repeated measures. The Kruskal–Wallis test (multigroup comparisons) was used for not normally distributed data with or without repeated measures. The Kruskal–Wallis test (multigroup comparisons) were used for data that were not normally distributed, with or without repeated measures. Experiments with two independent variables were analyzed by using two-way ANOVA or two-way RM-ANOVA if one of the variables was time. Šídák post hoc tests were applied where multiple comparisons were of interest. The significance level was set to *p* < 0.05 (significance levels denoted as follows: *p < 0.05; **p < 0.01; ***p < 0.001; ****p < 0.0001). All data are presented as means ± SEM unless stated otherwise.

## Results

### Impaired fear extinction in S1-DAT-Cre mice

**Figure 1.**
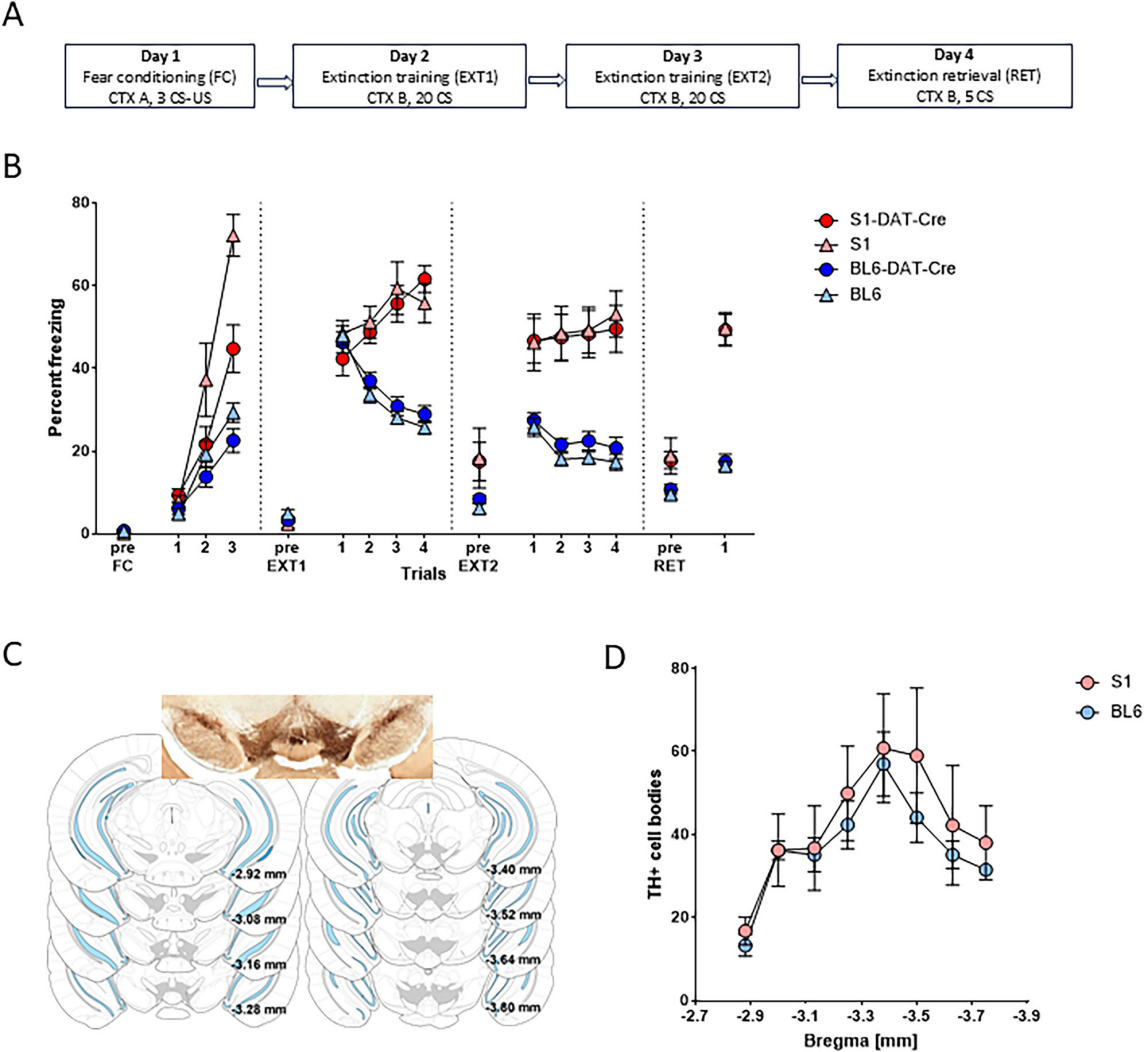
FC and extinction in S1-DAT-Cre, BL6-DAT-Cre, S1 and BL6 mice. ***A***, Experimental protocol for behavioral tests. ***B***, The percentage of time spent freezing during pre-CS periods or indicated CS presentations or blocks throughout the experimental paradigm. All groups increased freezing levels during FC. However, S1-DAT-Cre and S1 mice were freezing higher than BL6-DAT-Cre and BL6 mice during CS3, and also, S1 mice froze more than S1-DAT-Cre mice. At the beginning of the first extinction session (EXT1), all strains exhibited similar freezing levels. BL6 and BL6-DAT-Cre mice showed reduced freezing levels during the second extinction session (EXT2); S1 and S1-DAT-Cre mice maintained high freezing. During extinction retrieval (RET), S1-DAT-Cre and S1 mice again exhibited high freezing levels, as compared with BL6-DAT-Cre and BL6 mice. Note that S1 and S1-DAT-Cre mice overlapping data points on Extinction 2 (first-third data points) and retrieval (first block). S1-DAT-Cre (red circle), *n* = 12; S1 (pink triangle), *n* = 10; BL6-DAT-Cre (blue circle), *n* = 20; BL6 (light blue triangle), *n* = 22. Data are means ± SEM for the indicated time periods (pre-CS, single CS, or 5-CS average blocks). ***C***, Immunostaining for TH with representative image and atlas coordinates of cells counted relative to the bregma (S1 *n* = 7; BL6 *n* = 6). ***D***, Similar number of TH+ cell bodies in S1 and BL6 mice along the anterior–posterior axis. CTX, context; US, unconditioned stimulus; CS, conditioned stimulus. Data are means ± SEM for a section between the bregma −2.9 and −3.8 mm from the bregma. Extended Data Figure 1-1 supports this figure.

To enable viral targeting of VTA-DAergic neurons in the S1 mouse strain, we backcrossed DAT-Cre mice from a BL6 to S1 background for 9+ generations (F9). After backcrossing for 3–4 generations (F3/F4), S1-DAT-Cre mice were behaviorally tested to verify the line retained the extinction-impaired phenotype previously reported in S1 mice (Camp et al., 2009; Whittle et al., 2013). To this end, S1-DAT-Cre mice (*n* = 12) were compared with S1 (*n* = 10), BL6-DAT-Cre (*n* = 20), and BL6 mice (*n* = 22) in a tone-cue (conditioned stimulus, CS) pavlovian FC paradigm (FC, Day 1). Conditioning was followed by two consecutive daily fear extinction training sessions (EXT1 and EXT2, Days 2 and 3) and then an extinction retrieval test (RET; Day 4; see Materials and Methods for details; Fig. 1*A*).

10.1523/ENEURO.0174-25.2025.f1-1 Figure 1-1 **Statistical results.** ANOVA: analysis of variance, RM: repeated-measures, base: baseline, Veh: vehicle, YFP: yellow fluorescent protein, ChR2: channelrhodopsin2. For comparing two groups Student’s t-test (paired or unpaired) was used except where indicated. Data has normal distribution unless stated otherwise. Download Figure 1-1, DOCX file.

All groups increased freezing levels across CS presentations during FC, although to a different extent (two-way RM-ANOVA, “CS” *F*_(1,60)_ = 367.500; *p* < 0.0001; “strain” *F*_(3,60)_ = 24.830; *p* < 0.0001; interaction *F*_(3,60)_ = 28.470; *p* < 0.0001; Fig. 1*B*). All statistical analysis data can be found in Extended Data Figure 1-1. S1 mice showed significantly higher freezing levels than the other strains during the last CS presentation, and S1-DAT-Cre had higher freezing than BL6-DAT-Cre and BL6 mice (Šídák's post hoc tests adjusted *p* values, S1-DAT-Cre vs BL6-DAT-Cre, *p* < 0.0001; S1-DAT-Cre vs BL6, *p* = 0.0001; S1 vs BL6-DAT-Cre, *p* < 0.0001; S1 vs BL6, *p* < 0.0001; Fig. 1*B*).

The four strains exhibited similar freezing levels at the beginning of EXT1 (CS Block 1 = CS1–5; Šídák's post hoc tests, all *p* > 0.05). However, while BL6-DAT-Cre and BL6 animals reduced freezing levels across EXT1 (CS Block 1 to Block 4), S1-DAT-Cre and S1 mice did not (two-way RM-ANOVA, “CS block” *F*_(1,60)_ = 5.800; *p* = 0.0200; “strain” *F*_(3,60)_ = 11.300; *p* < 0.0001; interaction *F*_(3,60)_ = 55.200; *p* < 0.0001; Šídák's post hoc tests adjusted *p* values for CS Block 1 vs 4, S1-DAT-Cre *p* < 0.0001; S1 *p* = 0.1100; BL6-DAT-Cre *p* < 0.0001; BL6 *p* < 0.0001; Fig. 1*B*). Freezing levels remained higher in S1-DAT-Cre and S1 mice, as compared with BL6-DAT-Cre and BL6, throughout EXT2 (CS Block 1 to Block 4; two-way RM-ANOVA, “CS block” *F*_(1,60)_ = 1.160; *p* = 0.2900; “strain” *F*_(3,60)_ = 19.190; *p* < 0.0001; interaction *F*_(3,60)_ = 8.360; *p* < 0.0001; Šídák's post hoc tests adjusted *p* values for CS Block 1 vs 4, S1-DAT-Cre, *p* = 0.7700; S1, *p* = 0.1100; BL6-DAT-Cre, *p* = 0.0001; BL6, *p* < 0.0001) and RET (one-way ANOVA, *F*_(3,60)_ = 58.780; *p* < 0.0001; Šídák's post hoc tests adjusted *p* values, S1-DAT-Cre vs S1, *p* > 0.9900; BL6-DAT-Cre vs BL6, *p* = 0.9900; all other comparisons, *p* < 0.0001; Fig. 1*B*).

These behavioral data show that S1-DAT-Cre mice exhibit impaired fear extinction, comparable to S1 mice. On this basis, S1-DAT-Cre mice were used as a model to investigate the link between impaired extinction and VTA-DA neuronal function.

### Normal VTA-TH expression in S1-DAT-Cre mice

One possible explanation of the behavioral differences between S1-DAT-Cre and BL6-DAT-Cre mice could be strain differences in the number of VTA-DA containing neurons. Thus, VTA neurons in the two groups were immunohistochemically stained for TH and the number of TH-expressing neurons compared between the two strains (S1 *n* = 7; BL6 *n* = 6). This analysis found no significant group difference in the number of TH+ neurons (unpaired *t* test, *t*_(13.750)_ = 0.850; *p* = 0.4100; Fig. 1*C*,*D*). Hence, extinction differences between S1-DAT-Cre and BL6-DAT-Cre mice are unlikely to be due to overall differences in the number of VTA-DA containing neurons.

### Sustained extinction US omission-related VTA-DA neuronal responses in S1-DAT-Cre mice

**Figure 2.**
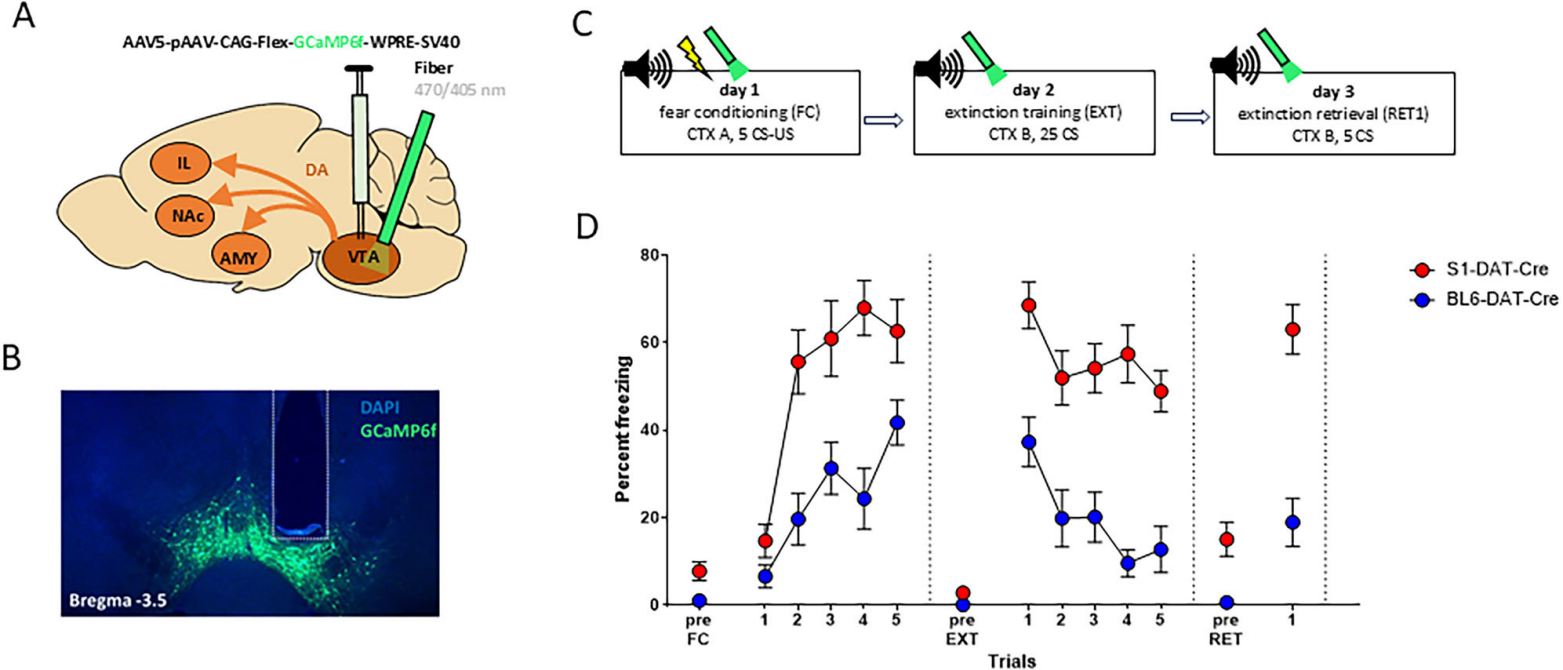
FC and extinction during fiber photometry. ***A***, AAV injection and implantation scheme. ***B***, Representative image of AAV expression and optic fiber tip locations in the VTA. ***C***, Experimental protocol for all behavioral tests. ***D***, The percentage of time spent freezing during pre-CS periods or indicated CS presentations or blocks throughout the experimental paradigm. Both strains showed increased freezing during FC, although S1-DAT-Cre mice exhibited generally higher freezing levels. During extinction training (EXT), freezing was decreased to a different extent in S1-DAT-Cre and BL6-DAT-Cre mice and S1-DAT-Cre mice showed more freezing than BL6-DAT-Cre mice during extinction retrieval (RET). CTX, context; US, unconditioned stimulus; CS, conditioned stimulus. Data are means ± SEM for the indicated time periods (pre-CS, single CS, or 5-CS average blocks). Extended Data Figures 1-1 and 2-1 support this figure.

To determine whether divergent fear extinction between S1-DAT-Cre and BL6-DAT-Cre mice is associated with variation in VTA-DA neuronal function, calcium (Ca^2+^) activity was measured in these neurons using in vivo fiber photometry. Mice had a viral construct containing the Ca^2+^ indicator, AAV5-pAAV-CAG-FLEX-GCaMP6f-WPRE-SV40 (S1-DAT-Cre, *n* = 9; BL6-DAT-Cre, *n* = 6) injected in the VTA and an optic fiber unilaterally chronically implanted above the injected area to measure GCaMP6 fluorescent signal during behavior (a, c). Representative image of injection site and fiber placement is shown in Figure 2*B*. For fiber tip placements, see Extended Data Figure 2-1. Mice underwent FC, EXT (25 times CS trials during one session) and RET testing, as described above.

10.1523/ENEURO.0174-25.2025.f2-1 Figure 2-1 **Fiber placement maps for fiber photometry experiments. A.** Optical fiber placements for the fiber photometry experiments with S1-DAT-Cre mice (CS-US; n=9). **B.** Optical fiber placements for the fiber photometry experiments with BL6-DAT-Cre mice (CS-US; n= 7). Download Figure 2-1, TIF file.

During FC, mice increased freezing levels (CS1–CS5) regardless of the group, although S1-DAT-Cre mice generally froze >BL6-DAT-Cre mice (two-way RM-ANOVA, “CS” *F*_(1,13)_ = 69.850; *p* < 0.0001; “strain” *F*_(1,13)_ = 5.620; *p* = 0.0339; “interaction” *F*_(1,13)_ = 1.650; *p* = 0.2212). There was a reduction in freezing levels during EXT (Block 1 to Block 5) in BL6-DAT-Cre and S1-DAT-Cre mice (two-way RM-ANOVA, “CS block” *F*_(1,13)_ = 49.910; *p* < 0.0001; “strain” *F*_(1,13)_ = 23.310; *p* = 0.0003; interaction *F*_(1,13)_ = 0.620; *p* = 0.4466; Fig. 2*D*). However, whereas BL6-DAT-Cre mice showed lower freezing on RET as compared with early EXT (Block 1), S1-DAT-Cre mice maintained high freezing levels (EXT Block 1 vs RET two-way RM-ANOVA, “CS block” *F*_(1,13)_ = 18.830; *p* = 0.0008; “strain” *F*_(1,13)_ = 24.040; *p* = 0.0003; interaction *F*_(1,13)_ = 5.440; *p* = 0.0363), which were higher than in BL6-DAT-Cre mice (unpaired *t* test, *t*_(13)_ = 5.330; *p* = 0.0001). These behavioral data (summarized in Fig. 2*D* with statistical data in Extended Data Fig. 1-1) confirm that S1-DAT-Cre mice exhibit an extinction memory-deficient phenotype during VTA-DA neuronal Ca^2+^ recordings.

**Figure 3.**
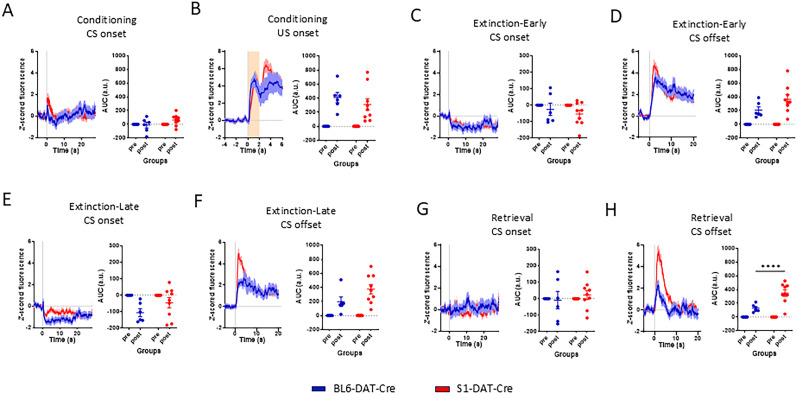
Fiber photometry during FC and extinction. Graphs show average *Z*-scores of changes in fluorescence (dF/*F*) during CS presentation, baselined to 5 s before CS onset or CS offset, along with corresponding time-normalized AUC values. ***A***, Fluorescence values during CS onset periods (0–5 s) of FC were higher in S1-DAT-Cre than in BL6-DAT-Cre mice. ***B***, Similarly increased fluorescence during US delivery and at US offset in S1-DAT-Cre and BL6-DAT-Cre animals. ***C***, During early EXT (First 10 CS trials), S1-DAT-Cre and BL6-DAT-Cre mice exhibited similar fluorescence at CS onset. ***D***, Similarly increased at early EXT CS offset in both strains. ***E***, During late EXT (last 10 CS trials), fluorescence at CS onset was less in BL6-DAT-Cre, but not in S1-DAT-Cre mice, as compared with early EXT. ***F***, Fluorescence at US omission at late EXT remained similarly high in both strains. ***G***, No change in fluorescence at CS onset during RET in either strain. ***H***, S1-DAT-Cre mice had higher fluorescence at US omission than BL6-DAT-Cre mice during RET. S1-DAT-Cre GCaMP6 (red), *n* = 9; BL6-DAT-Cre GCaMP6 (blue), *n* = 6. Data are means ± SEM. Extended Data Figures 1-1–3-3 support this figure.

Examination of calcium responses in VTA-DA neurons during FC showed S1-DAT-Cre mice exhibited similar activity at CS onset, as compared with BL6-DAT-Cre mice (two-way RM-ANOVA, “event” *F*_(1,13)_ = 1.090; *p* = 0.3154; “strain” *F*_(1,13)_ = 2.390; *p* = 0.1460; interaction *F*_(1,13)_ = 2.390; *p* = 0.1462; Šídák post hoc tests adjusted *p* values for BL6-DAT-Cre vs S1-DAT-Cre pre, *p* > 0.9999; post, *p* = 0.0745). Activity also increased in both strains at US onset (two-way RM-ANOVA, “event” *F*_(1,13)_ = 36.300; *p* < 0.0001; “strain” *F*_(1,13)_ = 0.690; *p* = 0.4223; interaction *F*_(1,13)_ = 0.690; *p* = 0.4226; Šídák post hoc tests adjusted *p* values for BL6-DAT-Cre vs S1-DAT-Cre pre, *p* > 0.999; post, *p* = 0.4406; Fig. 3*A*,*B*). These US-related responses resemble previous reports of VTA-DA neuronal activity during aversive and noxious events (Lammel et al., 2012; de Jong et al., 2019).

10.1523/ENEURO.0174-25.2025.f3-1 Figure 3-1 **Fiber photometry Ca^2+^ measurements in the VTA during CS/no-US fear conditioning.** All graphs show average *Z*-scores of changes in fluorescence (dF/F) for CS presentations baselined to 5 seconds before CS onset or CS offset and corresponding AUC values. **A**. There was no strain difference in fluorescence after CS onset during fear conditioning. **B**. Fluorescence at CS offset (2 seconds without US delivery) was similar between S1-DAT-Cre and BL6-DAT-Cre mice. **C**. During early EXT (First 10 CS trials), fluorescence at CS onset was not significantly different between strain. **D**. Fluorescent was not different between strains at US omission during early EXT. **E**. During late EXT (Last 10 CS trials), fluorescence at CS onset was not different between strains. **F**. During late EXT, fluorescence at US omission was similar across strains. **G and H.** Both strains no change in fluorescence at CS-onset and CS-offset during RET. S1-DAT-Cre GCaMP6 (red): n=4; BL6-DAT-Cre GCaMP6 (blue): n=3. Data are means ± SEM. Download Figure 3-1, TIF file.

10.1523/ENEURO.0174-25.2025.f3-2 Figure 3-2 **Fiber placement maps for fiber photometry experiments. A.** Optical fiber placements for the fiber photometry experiments with S1-DAT-Cre mice (CS-only; n=4). **B.** Optical fiber placements for the fiber photometry experiments with BL6-DAT-Cre mice (CS-only; n=3). Download Figure 3-2, TIF file.

10.1523/ENEURO.0174-25.2025.f3-3 Figure 3-3 **Fiber photometry Ca^2+^ measurements in the VTA during CS offset in Early Extinction and Retrieval.** All graphs show average *Z*-scores of changes in fluorescence (dF/F) for CS presentations baselined to 5 seconds before CS offset and corresponding AUC values. **A**. There was a significant difference in fluorescence at CS offset in BL6-DAT-Cre mice (n=6) on RET compared to early EXT (First 10 CS trials). **B**. Fluorescence at CS offset remained the same in S1-DAT-Cre mice (n=9) on RET compared to early EXT. Data are means ± SEM. Download Figure 3-3, TIF file.

During early EXT (first 10 CS trials), BL6-DAT-Cre and S1-DAT-Cre mice showed a modest but nonsignificant decrease in neuronal activity at CS onset (two-way RM-ANOVA, “event” *F*_(1,13)_ = 3.700; *p* = 0.0765; “strain” *F*_(1,13)_ = 0.440; *p* = 0.5191; interaction *F*_(1,13)_ = 0.440; *p* = 0.5165; Šídák post hoc tests adjusted *p* values for BL6-DAT-Cre vs S1-DAT-Cre pre, *p* > 0.999; post, *p* = 0.5850; Fig. 3*C*). In contrast, both strains showed a significant increase in activity at CS offset during early EXT, i.e., US omission (two-way RM-ANOVA, “event” *F*_(1,13)_ = 39.590; *p* < 0.0001; “strain” *F*_(1,13)_ = 3.030; *p* = 0.1053; interaction *F*_(1,13)_ = 3.020; *p* = 0.1057; Šídák post hoc tests adjusted *p* values for BL6-DAT-Cre vs S1-DAT-Cre pre, *p* > 0.9999; post, *p* = 0.0413; Fig. 3*D*). In a control experiment wherein mice were “conditioned” via presentation of the CS but not the US (CS-only), neuronal activity was not increased at CS offset at any test phase in either strain (see Extended Data Fig. 3-1 for data, Extended Data Fig. 3-2 for fiber tip placements, and Extended Data Fig. 1-1 for all statistical results).

During late EXT (last 10 CS trials), neuronal activity was again modestly reduced at CS onset in both strains, with a trend for a greater decrease in BL6-DAT-Cre mice (two-way RM-ANOVA, “event” *F*_(1,13)_ = 12.660; *p* = 0.0035; “strain” *F*_(1,13)_ = 1.960; *p* = 0.1847; interaction *F*_(1,13)_ = 1.970; *p* = 0.1844 Šídák post hoc tests adjusted *p* values for BL6-DAT-Cre vs S1-DAT-Cre pre, *p* > 0.9999; post, *p* = 0.1129; Fig. 3*E*). Activity at CS offset also remained elevated during late EXT for both strains, but now with a trend for higher activity in S1-DAT-Cre (two-way RM-ANOVA, “event” *F*_(1,13)_ = 32.870; *p* < 0.0001; “strain” *F*_(1,13)_ = 3.190; *p* = 0.0973; interaction *F*_(1,13)_ = 3.190; *p* = 0.0974; Šídák post hoc tests adjusted *p* values for BL6-DAT-Cre vs S1-DAT-Cre pre, *p* > 0.9999; post, *p* = 0.0356; Fig. 3*F*).

During RET, neuronal activity was not different on CS onset (Fig. 3*G*), but S1-DAT-Cre mice exhibited higher activity at CS offset, as compared with BL6-DAT-Cre mice (two-way RM-ANOVA, “event” *F*_(1,13)_ = 56.680; *p* < 0.0001; “strain” *F*_(1,13)_ = 12.230; *p* = 0.0039; interaction *F*_(1,13)_ = 12.220; *p* = 0.0040; Šídák post hoc tests adjusted *p* values for BL6-DAT-Cre vs S1-DAT-Cre pre, *p* > 0.9999; post, *p* < 0.0001; Fig. 3*H*). Notably, however, BL6-DAT-Cre mice exhibited lesser activity at CS offset on RET as compared with early EXT Block 1; S1-DAT-Cre did not show a significant change from early EXT to RET (two-way RM-ANOVA, “event” *F*_(1,5)_ = 24.840; *p* < 0.0042; “test” *F*_(1,5)_ = 8.331; *p* = 0.0343; interaction *F*_(1,5)_ = 8.345; *p* = 0.0342; Šídák post hoc tests adjusted *p* values for BL6-DAT-Cre Ext Block 1 vs RET pre, *p* > 0.9999; post, *p* = 0.0189; see Extended Data Figs. 3-3, 1-1 for all statistical results).

These data show, first, that successful extinction in BL6-background mice is associated with a PE-like response in VTA-DA neuronal activity at CS offset (i.e., omission of expected US) which is attenuated from early extinction to retrieval and, second, that the absence of this change in neuronal activity parallels the failure of S1-DAT-Cre mice to form an extinction memory.

### VTA-DA neuron photoexcitation during US omission does not rescue extinction in S1-DAT-Cre mice

**Figure 4.**
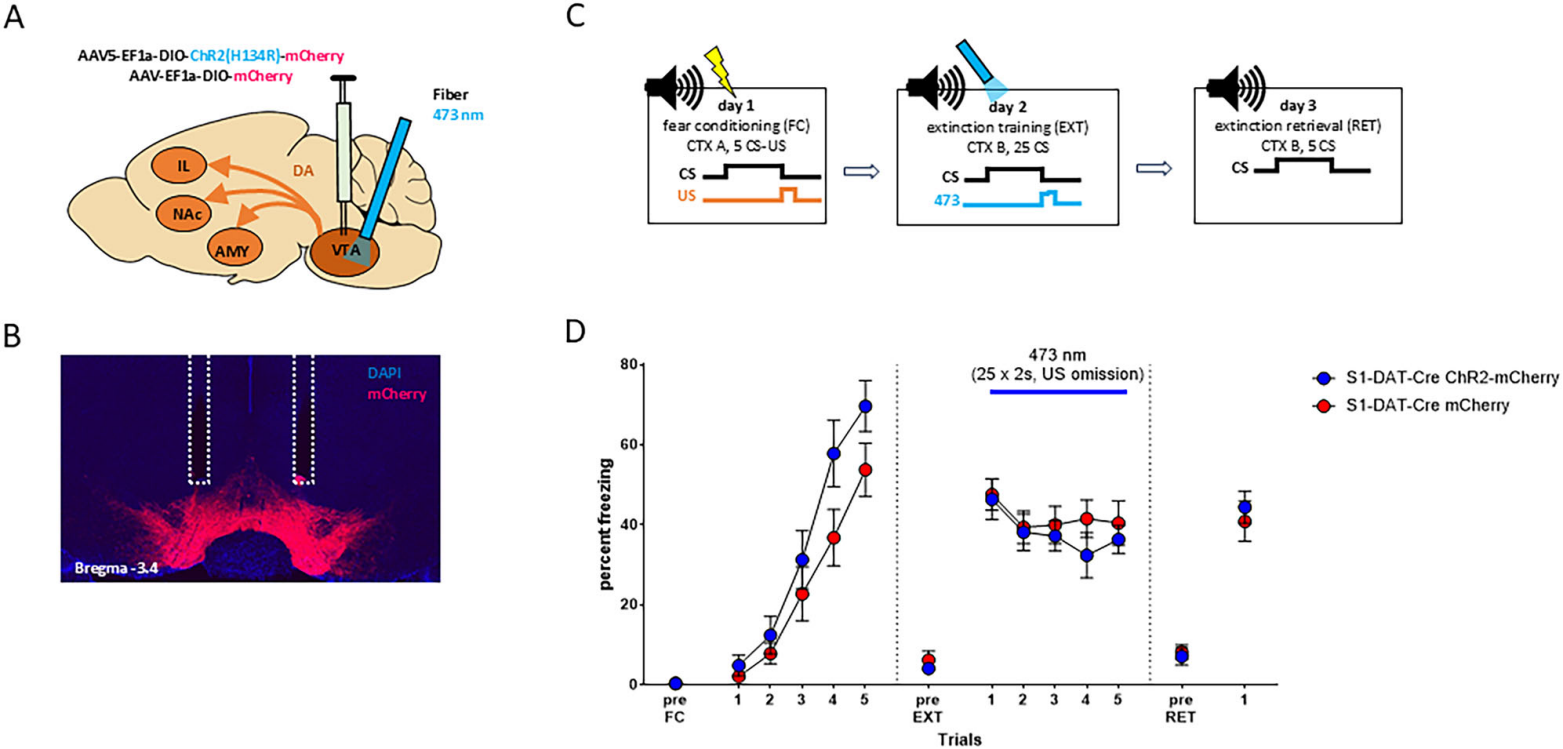
Optogenetic excitation of VTA-DAergic cell bodies during US omission in S1-DAT-Cre mice. ***A***, AAV injection and implantation scheme. ***B***, Representative image of AAV expression and optic fiber tip locations in the VTA. ***C***, Experimental protocol for all behavioral tests. ***D***, Freezing levels during pre-CS periods and indicated CS presentations or blocks. The opsin groups showed CS-related freezing during fear acquisition (FC). Optogenetic activation of VTA-DA neurons during US omission did not alter CS-related freezing during extinction training (EXT) or retrieval (RET). The opsin groups showed similar CS-related freezing levels during RET, as compared with the first CS block of extinction. S1-DAT-Cre ChR2-Cherry (blue), *n* = 12; S1-DAT-Cre mCherry (red), *n* = 10. CTX, context; US, unconditioned stimulus; CS, conditioned stimulus. Data are means ± SEM for the indicated time periods (pre-CS, single CS, or 5-CS average blocks). Extended Data Figures 1-1 and 4-1 support this figure.

One possible explanation for the sustained US omission-related VTA-DA neuronal activity in S1-DAT-Cre mice is that it is a compensatory, but insufficient, response to deficient DA signaling at extinction-mediating downstream target region(s). If so, experimentally augmenting activity at US omission might rescue deficient extinction in S1-DAT-Cre mice. To test this, a viral construct containing a Cre-dependent version of the excitatory opsin, channelrhodopsin [ChR2; AAV5-EF1a-DIO-ChR2(H134R)-mCherry; *n* = 10], or a control virus (AAV5-EF1a-DIO-mCherry; *n* = 11) was expressed in the VTA of S1-DAT-Cre mice, along with bilaterally chronically implanted optic fibers to direct 473 nm blue light at the VTA (Fig. 4*A*). Representative image of the injection site and fiber placement is shown in Figure 4*B*. For all fiber tip placements, see Extended Data Figures 4-1 and 1-1 for all statistical results. Mice were tested using the same behavioral protocol used for photometry but with the exception that light was delivered for 2 s at every US omission during EXT (Fig. 4*C*)—analogous to the optogenetic protocol previously shown to facilitate extinction in BL6-DAT-Cre mice (Salinas-Hernandez et al., 2018).

10.1523/ENEURO.0174-25.2025.f4-1 Figure 4-1 **Optrode placement maps for optogenetic experiments (VTA). A.** Optrode placements for the S1-DAT-Cre mCherry group shown in Figure 4 (n=10). **B.** Optrode placements for the S1-DAT-Cre ChR2 group shown in Figure 4 (n=12). Download Figure 4-1, TIF file.

The two virus groups showed comparable increases in freezing across CS trials (two-way RM-ANOVA for conditioning, “CS” *F*_(1,19)_ = 171.300; *p* < 0.0001; “opsin” *F*_(1,19)_ = 3.280; *p* = 0.090; interaction *F*_(1,19)_ = 2.19; *p* = 0.1500; summarized in Fig. 4*D*). During EXT + photoexcitation, there was a modest but significant reduction in freezing that was not different between the ChR2 and control virus group (two-way RM-ANOVA, “CS block” *F*_(1,19)_ = 8.270; *p* < 0.010; “opsin” *F*_(1,19)_ = 0.210; *p* = 0.6500; interaction *F*_(1,19)_ = 0.230; *p* = 0.6400). Likewise, freezing levels were statistically similar between the two groups during RET and were not different when compared with early EXT (Block 1; two-way RM-ANOVA, “CS block” *F*_(1,19)_ = 3.110; *p* = 0.0900; “opsin” *F*_(1,19)_ = 0.040; *p* = 0.8500; interaction *F*_(1,19)_ = 0.920; *p* = 0.3500). Note, one mouse from the S1-DAT-Cre ChR2-mCherry group that did not show robust freezing at early EXT (<25% mean freezing during CS Trials 1–2) was excluded from the statistical analysis.

The results of this optogenetic experiment suggest that augmenting VTA-DA neuronal activity is not sufficient to rescue impaired extinction in S1-DAT-Cre.

### VTA-DA→IL photoexcitation during US omission does not rescue extinction in S1-DAT-Cre mice

**Figure 5.**
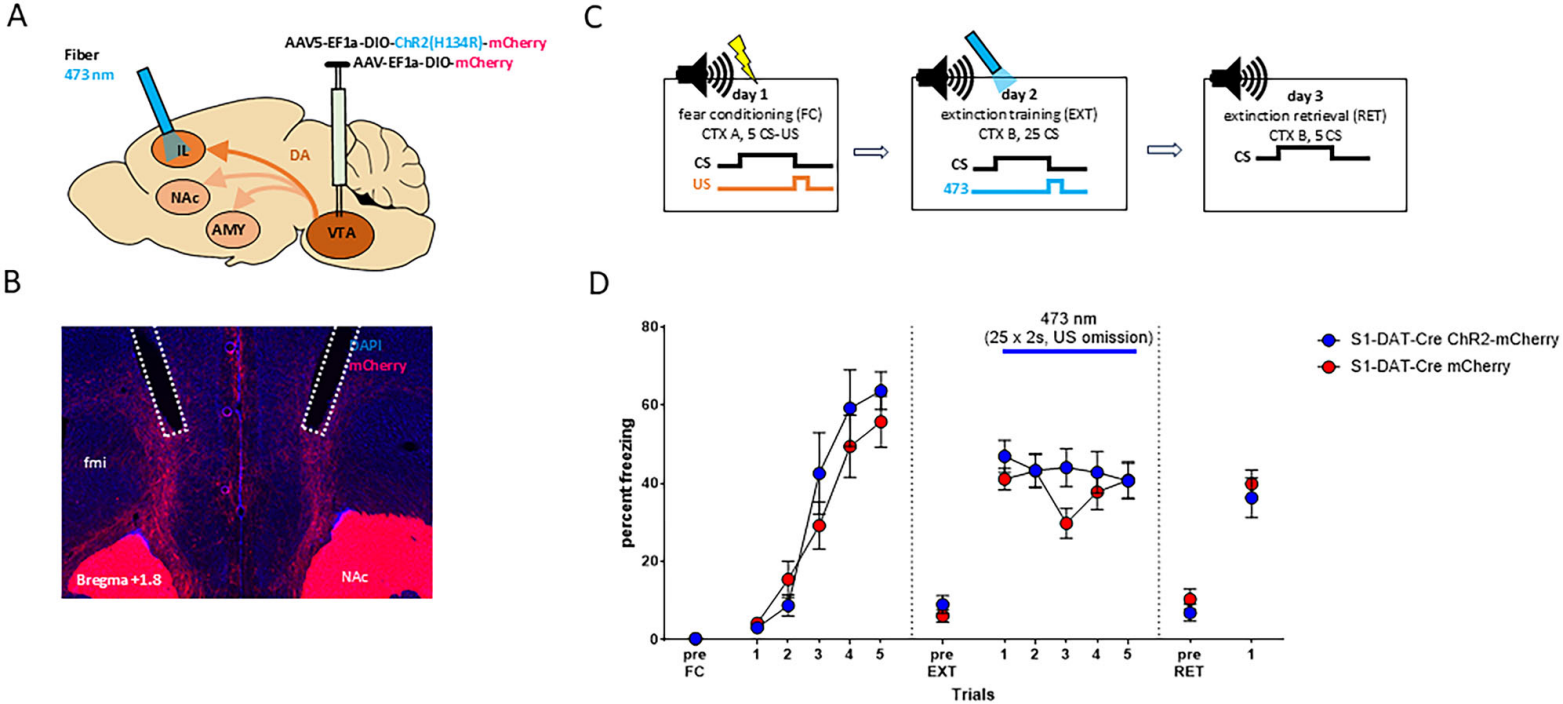
Optogenetic excitation of IL DAergic terminals during US omission in S1-DAT-Cre mice. ***A***, AAV injection and implantation scheme. ***B***, Representative image of AAV expression and optic fiber tip locations in the VTA. ***C***, Experimental protocol for all behavioral tests. ***D***, Freezing levels during pre-CS periods and indicated CS presentations or blocks. The opsin groups showed similar CS-related freezing during fear acquisition (FC). Optogenetic activation of VTA-DA axons in IL during US omission had no effect on CS-related freezing during extinction training (EXT) or retrieval (RET). The opsin groups showed similar freezing levels on RET, compared with the first CS block of extinction. S1-DAT-Cre ChR2-Cherry (blue), *n* = 9; S1-DAT-Cre mCherry (red), *n* = 11. CTX, context; US, unconditioned stimulus; CS, conditioned stimulus. Data are means ± SEM for the indicated time periods (pre-CS, single CS, or 5-CS average blocks). Extended Data Figures 1-1 and 5-1 support this figure.

One possible reason for the failure of VTA-DA neuronal photoexcitation to rescue extinction in S1-DAT-Cre mice is that VTA photoexcitation produced nonspecific increases in DA signaling at multiple forebrain target regions, some of which may impair extinction and oppose any extinction-facilitating effects at other regions. In this context, previous studies strongly implicate the IL in fear extinction (Maroun et al., 2012; Milad and Quirk, 2012; Bukalo et al., 2015, 2021; Do-Monte et al., 2015; Fitzgerald et al., 2015; Kim et al., 2016; Bloodgood et al., 2018; Lay et al., 2019; Gunduz-Cinar et al., 2023) and demonstrate a modulatory role of DA therein. For example, we recently reported that intra-IL infusion of DA rescues extinction in S1 mice (Sartori et al., 2024), and an fMRI study in humans demonstrated that activity patterns in the vmPFC (human analog of the IL) evident during extinction training spontaneously reappeared during postextinction rest, with the number of reactivations correlating with extinction memory strength (Gerlicher et al., 2018). Moreover, this relationship was amplified by postextinction systemic l-DOPA administration (Gerlicher et al., 2018), a drug which produces a short-term rescue of extinction in S1 mice (Whittle et al., 2016; Sartori et al., 2024). These cross-species observations led us to examine the effects of selectively targeting VTA neuronal axons in the IL. To do so, a Cre-dependent ChR2 virus (*n* = 9) or control virus (*n* = 11) was injected into the VTA of S1-DAT-Cre mice and bilaterally optic fibers chronically implanted in IL (for schematic, see Fig. 5*A*, and for representative image of injection site and fiber placement, see Fig. 5*B*) to photoexcite VTA-DA axons in IL during each US omission period during EXT (as above for VTA photoexcitation; Fig. 5*C*). For all fiber tip placements, see Extended Data Figure 5-1, and for all statistical results see Extended Data Figure 1-1.

10.1523/ENEURO.0174-25.2025.f5-1 Figure 5-1 **Optrode placement maps for optogenetic experiments (VTA-IL).** **A.** Optrode placements for the S1-DAT-Cre mCherry group shown in Figure 5 (n=11). **B.** Optrode placements for the S1-DAT-Cre ChR2 group shown in Figure 5 (n=9). Download Figure 5-1, TIF file.

During FC, freezing levels showed a comparable increase across CS trials in the two virus groups (two-way RM-ANOVA for conditioning CS1 compared with CS5 *F*_(1,18)_ = 159.500; *p* < 0.0100; “opsin” *F*_(1,18)_ = 0.640; *p* = 0.4400; interaction *F*_(1,18)_ = 1.050; *p* = 0.3200; Fig. 5*D*). When EXT was performed with concomitant VTA-DA→IL photoexcitation during US omission, neither group showed a decrease in freezing levels across CS blocks, and there was no group difference in freezing levels (two-way RM-ANOVA for CS Block1 compared with CS Block 5, “CS block” *F*_(1,18)_ = 0.990; *p* = 0.3300; “opsin” *F*_(1,18)_ = 0.340; *p* = 0.5600; interaction *F*_(1,18)_ = 0.850; *p* = 0.3700). The groups also showed similar freezing during RET, and freezing on RET was not different from early EXT (Block 1; two-way RM-ANOVA, “CS block” *F*_(1,18)_ = 3.440; *p* = 0.0800 “opsin” *F*_(1,18)_ = 0.070; *p* = 0.8000; interaction *F*_(1,18)_ = 2.140; *p* = 0.1600) and unpaired *t* test (*t*_(18)_ = 0.5817; *p* = 0.5700; Fig. 5*D*). Note, one mouse from the S1-DAT-Cre ChR2-mCherry group and two mice from the S1-DAT-Cre mCherry group did not show robust freezing during early EXT (<25% mean freezing during CS1–2) and were excluded from the statistical analysis.

The results of this experiment show that selective optogenetic photoexcitation of VTA-DA axons in IL during US omission was not sufficient to rescue impaired extinction in S1-DAT-Cre mice.

## Discussion

The role of midbrain DA signaling in fear extinction is currently under active investigation, yet the question of whether deficient DAergic signaling contributes to impairments in extinction remains to be fully elucidated. Using in vivo fiber photometry and optogenetics, the current experiments showed that VTA-DA neurons in extinction-impaired male S1-DAT-Cre mice and extinction-competent BL6-DAT-Cre mice exhibit increased activity at US omission during early extinction training—which represents a putative PE-like signal. This DA response changed with extinction (i.e., from early extinction training/fear retrieval to extinction retrieval) in BL6-DAT-Cre mice and was attenuated on extinction retrieval, similar to prior work in BL6-background mice (Salinas-Hernandez et al., 2018, 2023). In contrast, VTA-DA activity remained elevated across extinction training and retrieval in S1-DAT-Cre mice exhibiting impaired extinction. These data suggest that deficient extinction in S1 mice is not characterized by loss of putatively PE-like activity in VTA-DA neurons but instead is associated with the abnormal maintenance of this neuronal response.

Abnormalities in VTA-DA signaling and associated impairments in extinction in S1 background strain extend earlier studies on this inbred mouse strain. In addition to impaired extinction, S1 mice have been shown in different labs to exhibit other behavioral, including fear generalization (Camp et al., 2012; Temme et al., 2014), and neuroendocrine abnormalities relevant to PTSD (Hefner et al., 2008; Camp et al., 2009, 2012; Whittle et al., 2010, 2013; MacPherson et al., 2013; Cazares et al., 2019; Park and Chung, 2020; Fritz et al., 2021). Impaired extinction in these mice has also been linked to genetic variants (Gunduz-Cinar et al., 2019), and anomalous extinction-related neuronal features in the prefrontal cortex and amygdala (Fitzgerald et al., 2014; Gunduz-Cinar et al., 2019; O'Connor et al., 2019; Park and Chung, 2019, 2020; Whittle et al., 2021; Park et al., 2024). In addition, from a translational perspective, it is notable that extinction can be rescued in these mice by certain environmental and clinically relevant (e.g., chronic fluoxetine) pharmacological interventions (Whittle et al., 2009, 2010; Gunduz-Cinar et al., 2013, 2015; Sartori et al., 2016, 2024; Cazares et al., 2019).

The various phenotypic characteristics make this mouse a relevant model for studies examining the neural basis of fear extinction. It is worth noting, however, that in the current photometry experiment, S1 mice exhibited elevated freezing prior to the first CS presentation of FC and modest reductions in freezing during extinction training (although not retrieval). Given these patterns were not a consistent finding in other experiments here or in prior studies involving untethered mice (Camp et al., 2012; Gunduz-Cinar et al., 2019), the effect could reflect a motoric effect of tethering (e.g., fatigue) that was—for reasons to be determined—accentuated in this experiment. Given we have found that (BL6) mice become behaviorally equivalent to untethered controls with extensive habituation in other task settings entailing optical manipulations (Piantadosi et al., 2025), it would be interesting to test whether baseline freezing can be consistently normalized by such pretest experience.

Another important question for future work is the underlying cause of the abnormally sustained US omission-response in S1-DAT-Cre mice. Though immunohistochemical labeling found no strain difference in VTA-DA neuron number, the VTA contains subsets of neurons that are preferentially responsive to aversive and appetitive stimuli (de Jong et al., 2022). It therefore remains possible that the relative distribution of these functionally specialized subpopulations differs between the strains with, yet to be determined, consequences for fear extinction. Another possibility is that US omission-related VTA-DA neuronal activity is inadequately or improperly signaled to key extinction-mediating forebrain target regions. This could, in turn, result in the loss of a negative feedback response to the midbrain that normally drives extinction-related diminution of omission-related activity in VTA-DA neurons.

To augment potentially inadequate VTA-DA neuronal activity in S1-DAT-Cre mice, we employed in vivo optogenetics to photoexcite VTA-DA neurons or their axons in the IL during extinction US omission periods. Neither manipulation was sufficient to rescue extinction in these mice, at least with the optogenetic parameters used. The rationale for examining VTA-DA neuronal input to the IL was based on previous studies in humans and rodents linking DAergic actions in the IL to extinction (Maroun et al., 2012; Milad and Quirk, 2012; Bukalo et al., 2015, 2021; Do-Monte et al., 2015; Kim et al., 2016; Bloodgood et al., 2018; Lay et al., 2019; Gunduz-Cinar et al., 2023). For instance, several pharmacological studies also connect IL DA signaling with extinction (Singewald et al., 2015). Intra-IL administration of the D1R, D2R, or D4R antagonists either before or after extinction resulted in an impairment of extinction retrieval (Pfeiffer and Fendt, 2006; Hikind and Maroun, 2008; Mueller et al., 2010) and, in the case of D2R antagonism, attenuated CS-related responses in IL neurons (Mueller et al., 2010). Conversely, infusion of a D2R, but not D1R, agonist into the IL has been shown to facilitate extinction memory consolidation in adolescent animals (Zbukvic et al., 2017).

Using in vivo microdialysis, we recently showed that DA levels in the IL during extinction were similar in S1 and BL6 mice. However, multielectrode array recordings found reduced DA-evoked mPFC network responses in S1 mice (Sartori et al., 2024). Additionally, although mRNA expression of all DA receptor subtypes, as well as D1R and D2R binding, was similar between strains, D1R agonist-induced expression of a downstream effector of DA receptors, pERK, was lesser in the IL, but not PL, of S1 mice (Sartori et al., 2024). These data suggest that while extinction-related IL DA release is normal in S1 mice, abnormal downstream signaling might prevent DA from being conveyed into an effective extinction-mediating signal. Arguing against this explanation, however, intra-IL infusion of DA (Sartori et al., 2024) or systemic l-DOPA administration (Whittle et al., 2016) has been shown to rescue extinction in these mice.

One explanation for these apparently contradictory findings is that a sufficiently high level of IL DA is required to overcome downstream signaling abnormalities and produce consequent behavioral effects in S1 mice. Direct DA infusion would be more likely to produce this effect than axonal photoexcitation particularly if—although this remains to be determined—there is lesser VTA-DA neuronal innervation of the IL in S1 than BL6 mice. To clarify these findings, it would be valuable to measure US omission-related activity in VTA-DA axons in the IL and to verify the ability of photoexcitation to increase DA levels in the IL of these mice. It would also be informative to measure extinction-related release of IL DA in S1 mice from sources other than the VTA, including the locus ceruleus (Devoto and Flore, 2006; Devoto et al., 2020). Such work would be particularly valuable given we do not provide technical confirmation that optogenetic photoexcitation of VTA neurons or VTA-IL fibers effectively modulated DA activity and release.

Of note in this context, a prior study found that optogenetic photosilencing of VTA-DA neuronal axons in the IL during extinction US omission periods improved extinction retrieval in rats (Luo et al., 2018). IL-projecting VTA-DA neurons have also been shown to signal aversion (Lammel et al., 2011, 2012; Vander Weele et al., 2018; Weele et al., 2019). These findings suggest that this pathway opposes, rather than facilitates, extinction memory formation, offering another potential explanation for our negative optogenetic data. They also raise the corollary question of whether photoexcitation of other VTA-DA neuronal targets would promote extinction. In this regard, VTA-DA inputs to the (medial) nucleus accumbens (NAc) exhibit an extinction PE in extinction-competent rodents (Salinas-Hernandez et al., 2018, 2023; Cai et al., 2020), and optogenetic photosilencing of this pathway impairs extinction formation in BL6 mice (Salinas-Hernandez et al., 2023) and extinction consolidation in TH-Cre rats (Luo et al., 2018). Another recent study suggested the NAc shell, rather than core, could potentially be the key site for these effects (Dutta et al., 2021). These rodent data may have clinical relevance given US omission activation in the human analog of the NAc, the ventral striatum, which was recently associated with extinction efficacy and VTA activity, in a manner that is enhanced by l-DOPA administration (Raczka et al., 2011; Esser et al., 2021).

In summary, the current study provides novel evidence of altered extinction-related VTA-DA neuronal dynamics in a mouse model of impaired fear extinction and, furthermore, finds that optogenetically exciting VTA-DA neurons or their inputs to the IL is insufficient to rescue this impairment. It should be noted, however, that these findings were all made using male mice and whether they generalize to female mice remains an important question for future work. Nevertheless, given fear extinction is a translationally tractable process with an increasingly well-defined neural circuit basis (Herry et al., 2010; Arruda-Carvalho and Clem, 2015; Aksoy-Aksel et al., 2025), the current findings in mice could have implications for understanding the neural basis of impaired extinction in various neuropsychiatric disorders.

## Data Availability

Data will be made available upon reasonable request to the corresponding author.

## Synthesis

Reviewing Editor: Jibran Khokhar, Western University Department of Anatomy and Cell Biology Decisions are customarily a result of the Reviewing Editor and the peer reviewers coming together and discussing their recommendations until a consensus is reached. When revisions are invited, a fact-based synthesis statement explaining their decision and outlining what is needed to prepare a revision will be listed below. The following reviewer(s) agreed to reveal their identity: Gunes Kutlu, Laura Bradfield.

Summary

This manuscript explores the role of ventral tegmental area (VTA) dopamine (DA) neurons in fear extinction using a mouse model (129S1/SvImJ or S1) known for impaired extinction. Through fiber photometry and optogenetic manipulations, the authors aim to identify neural correlates and potential interventions. The study offers a potentially important contribution by examining dopaminergic dynamics in a model of extinction impairment and by attempting circuit-based rescue approaches. However, both reviewers note that the data are overinterpreted in several places, with conclusions extending beyond what is statistically supported. The reviewers agree the work is well-structured and that the publication of null results is still valuable, but they recommend substantial revisions to temper the interpretations and provide clarification on methodology and model characterization.

Major Points

Overinterpretation of Fiber Photometry Results

The authors frequently draw conclusions about group differences and within-group effects in VTA-DA activity that are not statistically supported. Only one clear between-group difference is reported (Figure 3H, CS offset during retrieval). All other inferences, particularly those regarding differences during extinction, should be removed or appropriately qualified. Any claims of prediction error-like signaling must be backed by formal within-group comparisons across extinction phases.

Questionable Interpretation of S1 Phenotype as Extinction-Specific

The behavioral data suggest that S1 mice may simply exhibit heightened generalized fear, not a specific deficit in extinction memory. Differences in freezing precede extinction training (notably in Figure 2), weakening claims of an extinction-specific impairment. The authors should consider and discuss whether the phenotype reflects increased baseline fear rather than an isolated extinction deficit.

Uncertain Efficacy of Optogenetic Manipulation

The manuscript does not provide sufficient evidence that optogenetic stimulation of VTA-DA neurons or IL terminals effectively modulated DA activity. Without electrophysiological, immunohistochemical (e.g., c-Fos), or behavioral evidence of engagement, it is unclear whether the lack of rescue reflects a true null effect or a technical failure. This possibility must be acknowledged, and language around the insufficiency of stimulation should be qualified (e.g., "with the parameters used").

Discrepancies Across Figures and Inconsistent Behavioral Effects

There is inconsistency in extinction curve shapes across Figures 1, 2, and 3-5. In particular, extinction appears less impaired in Figure 2 than in Figure 1, and this inconsistency is not addressed. Differences in trial averaging or baseline freezing levels may explain this and should be clarified.

Need for Greater Rigor in Statistical Reporting and Interpretation

Across several figures, claims are made without supporting statistical results. For example, Figure 3C and 3E are discussed as if group differences exist, despite no significant strain or interaction effects. The text and figure legends need to be revised to match the statistical findings precisely, particularly in distinguishing observed trends from supported results.

Minor Points

Characterization of the S1 Model

More background is needed on the S1 mouse strain, its known phenotypes, genetic characteristics, and why it is relevant to extinction research. This is essential for readers unfamiliar with this model.

Sex of Mice and Generalizability

Only male mice were used, but this is neither mentioned in the abstract nor addressed in the discussion. A disclaimer should be added regarding limitations in generalizability to females.

Clarification of Data Visualization (Figure 1B)

In the extinction retrieval plot of Figure 1B, it is unclear whether points from the S1 and S1-DAT-Cre groups are overlapping. This should be clarified to improve interpretability.

Consistency in Figure Referencing

Figure numbers should be placed immediately after the relevant claims in the text, rather than grouped at the end of paragraphs, to aid readability and reference clarity.
