# Supplementary material for: Altered Dopamine Signaling in Extinction-Deficient Mice
Source: eNeuro. 2025 Nov 18;12(11):ENEURO.0174-25.2025. doi: 10.1523/ENEURO.0174-25.2025 (PMC12658411; doi:10.1523/ENEURO.0174-25.2025)
Supplement: Figure 1-1 — Statistical results. ANOVA: analysis of variance, RM: repeated-measures, base: baseline, Veh: vehicle, YFP: yellow fluorescent protein, ChR2: channelrhodopsin2. For comparing two groups Student’s t-test (paired or unpaired) was used except where indicated. Data has normal distribution unless stated otherwise. Download Figure 1-1, DOCX file. [file eneuro-12-ENEURO.0174-25.2025-s002.docx]

| **Data shown in Figure 1** | | | | | | |
| --- | --- | --- | --- | --- | --- | --- |
| **Strain comparison for behavior (Figure 1b)** | | | | | | |
| *Strain Comparison* | *Statistical test* | | *Trial effect* | *Strain effect* | | *Interaction* |
| Conditioning trial 1 vs trial 3 | 2-factor ANOVA  (RM for Trial) | | *F*(1,60)=367.500, p*<0.0001* | *F*(3,60)=24.830, p<0.0001 | | *F*(3,60)=28.470, p<0.0001 |
| Šídák’s tests: S1-DAT-Cre vs BL6-DAT-Cre: p<0.0001, S1-DAT-Cre vs BL6: p=0.0001, S1 vs BL6-DAT-Cre: p<0.0001, S1 vs. BL6: p<0.0001 | | | | | | |
| Extinction 1 block 1 vs block 4 | 2-factor ANOVA  (RM for Trial) | | *F*(1,60)=5.800, p*=*0.0191 | *F*(3,60)=11.300, p<0.0001 | | *F*(3,60)=55.200, p<0.0001 |
| Šídák’s tests: S1-DAT-Cre: p<0.0001, S1: p=0.11, BL6-DAT-Cre: p<0.0001, BL6: p<0.0001 | | | | | | |
| Extinction 2 block 1 vs block 4 | 2-factor ANOVA  (RM for Trial) | | *F*(1,60)=1.160, p*=*0.2852 | *F*(3,60)=19.190, p<0.0001 | | *F*(3,60)=8.360, p<0.0001 |
| Šídák’s tests: S1-DAT-Cre: p=0.77, S1: p=0.11, BL6-DAT-Cre: p*=*0.01, BL6: p*<*0.0001 | | | | | | |
| Retrieval | Ordinary 1 factor ANOVA | | - | *F*(3,60)=58.780, p<0.0001 | | - |
| Šídák’s tests: S1-DAT-Cre vs S1: p>0.99, S1-DAT-Cre vs BL6-DAT-Cre: p=0.99, S1-DAT-Cre vs BL6: p<0.0001, S1 vs BL6-DAT-Cre: p<0.0001, S1 vs BL6: p<0.0001, BL6-DAT-Cre vs BL6: p<0.0001 | | | | | | |
| **Stereology S1 vs BL6 (Figure 1d)** | | | | | | |
| *Measure/Comparison* | | *Statistical test* | | | *Result* | |
| TH+ cell counts | | Unpaired t-test | | | *t*(13.75)=0.845, p=0*.*4125 | |
| **Data shown in Figure 2** | | | | | | |
| **GCAMP6m recordings behavior for S1-DAT-Cre vs BL6-DAT-Cre (Figure 2d)** | | | | | | |
| *Strain Comparison* | *Statistical test* | | *Event effect* | *Strain effect* | | *Interaction* |
| Conditioning (all) | 2-factor ANOVA  (RM for Trial) | | *F*(5,65)=27.950, p*<*0.0001 | *F*(1,13)=20.250, p=0.0006 | | *F*(5,65)=3.820, p=0.0043 |
| Šídák’s tests: S1-DAT-Cre vs BL6-DAT-Cre baseline: p=0.9716, CS 1: p=0.9321, CS 2: p=0.0006, CS 3: p=0.0072, CS 4: p<0.0001, CS 5: p=0.1164 | | | | | | |
| Conditioning CS1 vs CS5 | 2-factor ANOVA  (RM for Trial) | | *F*(1,13)=69.850, p*<*0.0001 | *F*(1,13)=5.620, p=0.0339 | | *F*(1,13)=1.650, p=0.2212 |
| Šídák’s tests: S1-DAT-Cre vs BL6-DAT-Cre CS 1: p=0.5286, CS 5: p=0.0269 | | | | | | |
| Extinction block 1 vs block 5 | 2-factor ANOVA  (RM for Trial) | | *F*(1,13)=49.910, p*<*0.0001 | *F*(1,13)=23.310, p=0.0003 | | *F*(1,13)=0.620, p=0.4466 |
| Šídák’s tests: S1-DAT-Cre vs BL6-DAT-Cre block 1: p=0.0007, block 5: p=0.0001 | | | | | | |
| Retrieval | Unpaired t-test | | *t*(13)=5.330, p=0.0001 | | | |
| Extinction block 1 vs Retrieval block | 2-factor ANOVA  (RM for Trial) | | *F*(1,13)=18.830, p*=*0.0008 | *F*(1,13)=24.040, p=0.0003 | | *F*(1,13)=5.440, p=0.0363 |
| Šídák’s tests: Extinction block 1 vs Retrieval block: S1-DAT-Cre p=0.2550, BL6-DAT-Cre: p=0.0017 | | | | | | |
| **Data shown in Figure 3** | | | | | | |
| **S1-DAT Cre vs BL6-DAT Cre for CS-US comparison** | | | | | | |
| **Conditioning (Figure 3a, b)** | | | | | | |
| *Strain Comparison* | *Statistical test* | | *Event effect* | *Strain effect* | | *Interaction* |
| Conditioning CS onset | 2-factor ANOVA  (RM for Trial) | | *F*(1,13)=1.090, p*=*0.3154 | *F*(1,13)=2.390, p=0.1460 | | *F*(1,13)=2.390, p=0.1462 |
| Šídák’s tests: S1-DAT-Cre vs BL6-DAT-Cre pre: p>0.9999, post: p=0.0745 | | | | | | |
| *Strain Comparison* | *Statistical test* | | *Event effect* | *Strain effect* | | *Interaction* |
| Conditioning CS offset | 2-factor ANOVA  (RM for Trial-block) | | *F*(1,13)=53.720, p<0.0001 | *F*(1,13)=0.300, p=0.5950 | | *F*(1,13)=0.300, p=0.5953 |
| Šídák’s tests: S1-DAT-Cre vs BL6-DAT-Cre pre: p>0.999, post: p=0.6953 | | | | | | |
| Conditioning US onset | 2-factor ANOVA  (RM for Test-phase) | | *F*(1, 13)=36.300, p<0.0001 | *F*(1, 13)=0.690, p=0.4223 | | *F*(1, 13)=0.690, p=0.4226 |
| Šídák’s tests: S1-DAT-Cre vs BL6-DAT-Cre pre: p>0.999, post: p=0.4406 | | | | | | |
| *Strain Comparison* | *Statistical test* | | *Event effect* | *Strain effect* | | *Interaction* |
| Conditioning US onset | *ANOVA with Kruskal-Wallis test (preS1 group didn’t pass normality test)* | | *-* | *H(3)=22.300,* p<0.0001 | | *-* |
| **Extinction (Figure 3c-f)** | | | | | | |
| Early extinction (First 10 CS) CS onset | 2-factor ANOVA  (RM for Test-phase) | | *F*(1, 13)=3.700, p=0.0765 | *F*(1, 13)=0.440, p=0.5191 | | *F*(1, 13)=0.440, p=0.5165 |
| Šídák’s tests: S1-DAT-Cre vs BL6-DAT-Cre pre: p>0.999, post: p=0.5850 | | | | | | |
| Early extinction (First 10 CS) CS offset | 2-factor ANOVA  (RM for Test-phase) | | *F*(1, 13)=39.590, p<0.0001 | *F*(1, 13)=3.030, p=0.1053 | | *F*(1, 13)=3.020, p=0.1057 |
| Šídák’s tests: S1-DAT-Cre vs BL6-DAT-Cre pre: p>0.9999, post: p=0.0413 | | | | | | |
| Late extinction (Last 10 CS) CS onset | 2-factor ANOVA  (RM for Test-phase) | | *F*(1, 13)=12.660, p=0.0035 | *F*(1, 13)=1.960, p=0.1847 | | *F*(1, 13)=1.970, p=0.1844 |
| Šídák’s tests: S1-DAT-Cre vs BL6-DAT-Cre pre: p>0.9999, post: p=0.1129 | | | | | | |
| Late extinction (Last 10 CS) CS offset | 2-factor ANOVA  (RM for Test-phase) | | *F*(1, 13)=32.870, p<0.0001 | *F*(1, 13)=3.190, p=0.0973 | | *F*(1, 13)=3.190, p=0.0974 |
| Šídák’s tests: S1-DAT-Cre vs BL6-DAT-Cre pre: p>0.9999, post: p=0.0356 | | | | | | |
| **Retrieval (Figure 3g,h)** | | | | | | |
| Retrieval CS onset | 2-factor ANOVA  (RM for Test-phase) | | *F*(1, 13)=0.050, p=0.8292 | *F*(1, 13)=0.320, p=0.5825 | | *F*(1, 13)=0.320, p=0.5831 |
| Šídák’s tests: S1-DAT-Cre vs BL6-DAT-Cre pre: p>0.9999, post: p=0.6784 | | | | | | |
| Retrieval CS offset | 2-factor ANOVA  (RM for Test-phase) | | *F*(1, 13)=56.680, p<0.0001 | *F*(1, 13)=12.230, p=0.0039 | | *F*(1, 13)=12.220, p=0.0040 |
| Šídák’s tests: S1-DAT-Cre vs BL6-DAT-Cre pre: p>0.9999, post: p<0.0001 | | | | | | |
| **Early extinction (First 10 CS) CS offset vs Retrieval offset (Extended Figure 3-3)** | | | | | | |
| Comparison for |  | | Event | Test | | Interaction |
| BL6-DAT-Cre | 2-factor ANOVA  (RM for Test-phase) | | *F*(1, 5)=24.840, p<0.0042 | *F*(1, 5)=8.331, p=0.0343 | | *F*(1, 5)=8.345, p=0.0342 |
| Šídák post-hoc tests adjusted p-values for BL6-Dat-Cre Ext CS block 1 vs Retrieval pre: p>0.9999, post: p=0.0189 | | | | | | |
| S1-DAT-Cre | 2-factor ANOVA  (RM for Test-phase) | | *F*(1, 8)=56.950, p<0.0001 | *F*(1, 8)=0.056, p=0.8181 | | *F*(1, 8)=0.056, p=0.8184 |
| Šídák post-hoc tests adjusted p-values for S1-Dat-Cre Ext CS block 1 vs Retrieval pre: p>0.9999, post: p=0.9353 | | | | | | |
| **Data shown in Extended Figure 3-1** | | | | | | |
| **S1-DAT Cre vs BL6-DAT Cre for CS only comparison** | | | | | | |
| **Conditioning (Extended figure 3-1 a,b)** | | | | | | |
| *Strain Comparison* | *Statistical test* | | *Event effect* | *Strain effect* | | *Interaction* |
| Conditioning CS onset | 2-factor ANOVA  (RM for Trial) | | *F*(1,5)=0.020, p*=*0.9046 | *F*(1,5)=2.370, p=0.1847 | | *F*(1,5)=2.360, p=0.1847 |
| Šídák’s tests: S1-DAT-Cre vs BL6-DAT-Cre pre: p>0.999, post: p=0.1065 | | | | | | |
| Conditioning CS offset | 2-factor ANOVA  (RM for Trial) | | *F*(1,5)=1.140, p*=*0.3344 | *F*(1,5)=0.070, p=0.8054 | | *F*(1,5)=0.070, p=0.8070 |
| Šídák’s tests: S1-DAT-Cre vs BL6-DAT-Cre pre: p>0.99, post: p=0.9228 | | | | | | |
| **Extinction (Extended figure 3-1 c-f)** | | | | | | |
| Early extinction CS onset | 2-factor ANOVA  (RM for Trial) | | *F*(1,5)=1.360, p*=*0.2961 | *F*(1,5)=3.310, p=0.1285 | | *F*(1,5)=3.310, p=0.1284 |
| Šídák’s tests: S1-DAT-Cre vs BL6-DAT-Cre pre: p>0.99, post: p=0.0547 | | | | | | |
| Early extinction CS offset | 2-factor ANOVA  (RM for Trial) | | *F*(1,5)=5.270, p*=*0.0702 | *F*(1,5)=0.010, p=0.9873 | | *F*(1,5)=0.010, p=0.9869 |
| Šídák’s tests: S1-DAT-Cre vs BL6-DAT-Cre pre: p>0.9999, post: p=0.9997 | | | | | | |
| Late extinction CS onset | 2-factor ANOVA  (RM for Trial) | | *F*(1,5)=0.500, p*=*0.5129 | *F*(1,5)=13.280, p=0.0148 | | *F*(1,5)=13.190, p=0.0150 |
| Šídák’s tests: S1-DAT-Cre vs BL6-DAT-Cre pre: p>0.9999, post: p=0.0009 | | | | | | |
| *Strain Comparison* | *Statistical test* | | *Event effect* | *Strain effect* | | *Interaction* |
| Late extinction CS onset | *ANOVA with Kruskal-Wallis test (post BL6 group didn’t pass normality test)* | | *-* | *H(3)=11.130,* p=0.0002 | | *-* |
| Late extinction CS offset | 2-factor ANOVA  (RM for Trial) | | *F*(1,5)=5.470, p=0.0665 | *F*(1,5)=1.685, p=0.2509 | | *F*(1,5)=1.690, p=0.2503 |
| Šídák’s tests: S1-DAT-Cre vs BL6-DAT-Cre pre: p>0.9999, post: p=0.1829 | | | | | | |
| **Retrieval (Extended figure 3-1 g,h)** | | | | | | |
| Retrieval CS onset | 2-factor ANOVA  (RM for Trial) | | *F*(1,5)=4.330, p=0.0919 | *F*(1,5)=0.040, p=0.8525 | | *F*(1,5)=0.040, p=0.8536 |
| Šídák’s tests: S1-DAT-Cre vs BL6-DAT-Cre pre: p>0.99, post: p=0.9552 | | | | | | |
| Retrieval CS offset | 2-factor ANOVA  (RM for Trial) | | *F*(1,5)=0.010, p=0.9652 | *F*(1,5)=0.410, p=0.5480 | | *F*(1,5)=0.410, p=0.5490 |
| Šídák’s tests: S1-DAT-Cre vs BL6-DAT-Cre pre: p>0.99, post: p=0.6212 | | | | | | |
| *Strain Comparison* | *Statistical test* | | *Event effect* | *Strain effect* | | *Interaction* |
| Retrieval CS offset | *ANOVA with Kruskal-Wallis test (post BL6 group didn’t pass normality test)* | | *-* | *H(3)=0.733,* p=0.8902 | | *-* |
| **Data shown in Figure 4** | | | | | | |
| **VTA-DA neuron photoexcitation during US omission in S1-DAT-Cre mice (Figure 4d)** | | | | | | |
| *Strain Comparison* | *Statistical test* | | *Trial effect* | *Opsin effect* | | *Interaction* |
| Conditioning trial 1 vs trial 5 | 2-factor ANOVA  (RM for Trial) | | *F*(1,19)=2.186, p=0.16 | *F*(1,19)=3.280, p=0.086 | | *F*(1,19)=2.186, p=0.15 |
| Šídák’s tests: ChR2-mCherry vs mCherry: CS1: p=0.90, CS5: p=0.05 | | | | | | |
| Extinction 1 block 1 vs block 5 | 2-factor ANOVA  (RM for Trial) | | *F*(1,19)=8.268, p*=*0.097 | *F*(1,19)=0.207, p=0.6537 | | *F*(1,19)=0.232, p*=*0.6351 |
| Šídák’s tests: ChR2-mCherry vs mCherry: CS block1: p=0.9785, CS block 5: p=0.7842 | | | | | | |
| Extinction 1 block 1 vs Retrieval block 1 | 2-factor ANOVA  (RM for Trial) | | *F*(1,19)=3.108, p*=*0.094 | *F*(1,19)=0.037, p=08487 | | *F*(1,19)=0.923, p*=*0.3485 |
| Šídák’s tests: ChR2-mCherry vs mCherry: Extinction CS block1: p=0.9780, Retrieval CS block 1: p=0.8305 | | | | | | |
| Retrieval | Unpaired t-test | | *t*(19)=0.538, p=0.5967 | | | |
| **Data shown in Figure 5** | | | | | | |
| **VTA-DA→IL photoexcitation during US omission in S1-DAT-Cre mice (Figure 5d)** | | | | | | |
| *Strain Comparison* | *Statistical test* | | *Trial effect* | *Opsin effect* | | *Interaction* |
| Conditioning trial 1 vs trial 5 | 2-factor ANOVA  (RM for Trial) | | *F*(1,18)=159.500, p<0.0001 | *F*(1,18)=0.636, p=0.4355 | | *F*(1,18)=1.053, p=0.3184 |
| Šídák’s tests: ChR2-mCherry vs mCherry: CS1: p=0.98, CS5: p=0.37 | | | | | | |
| Extinction 1 block 1 vs block 5 | 2-factor ANOVA  (RM for Trial) | | *F*(1,18)=0.990, p*=*0.3316 | *F*(1,18)=0.345, p*=*0.5644 | | *F*(1,18)=0.847, p*=*0.3695 |
| Šídák’s tests: ChR2-mCherry vs mCherry: CS block1: p=0.5388, CS block 5: p=0.999 | | | | | | |
| Extinction 1 block 1 vs Retrieval block 1 | 2-factor ANOVA  (RM for Trial) | | *F*(1,18)=3.437, p*=*0.080 | *F*(1,18)=0.067, p=0.798 | | *F*(1,18)=2.136, p*=*0.1611 |
| Šídák’s tests: ChR2-mCherry vs mCherry: Extinction CS block1: p=0.5020, Retrieval CS block 1: p=0.7727 | | | | | | |
| Retrieval | Unpaired t-test | | *t*(18)=0.582, p=0.57 | | | |
