# Supplementary figures and images for: Altered Dopamine Signaling in Extinction-Deficient Mice
Source: eNeuro. 2025 Nov 18;12(11):ENEURO.0174-25.2025. doi: 10.1523/ENEURO.0174-25.2025 (PMC12658411; doi:10.1523/ENEURO.0174-25.2025)

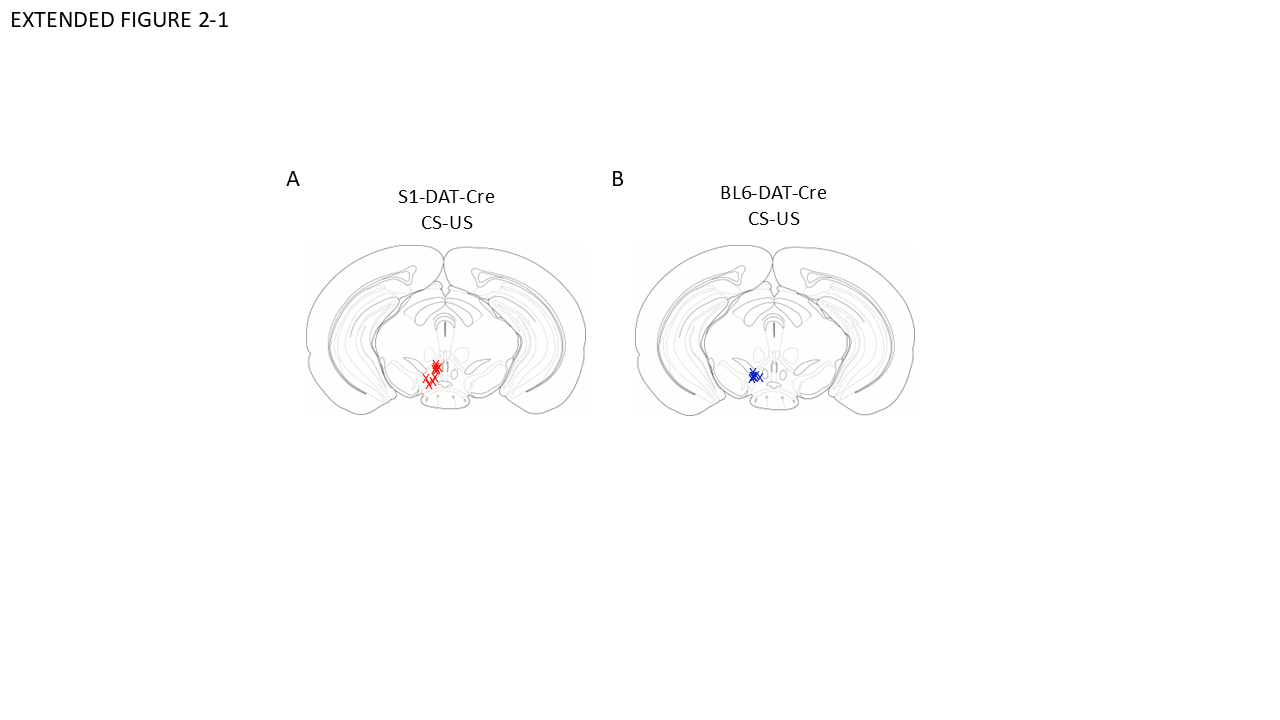

Supplement: Figure 2-1 — Fiber placement maps for fiber photometry experiments. A. Optical fiber placements for the fiber photometry experiments with S1-DAT-Cre mice (CS-US; n=9). B. Optical fiber placements for the fiber photometry experiments with BL6-DAT-Cre mice (CS-US; n= 7). Download Figure 2-1, TIF file. [file eneuro-12-ENEURO.0174-25.2025-s003.tif]

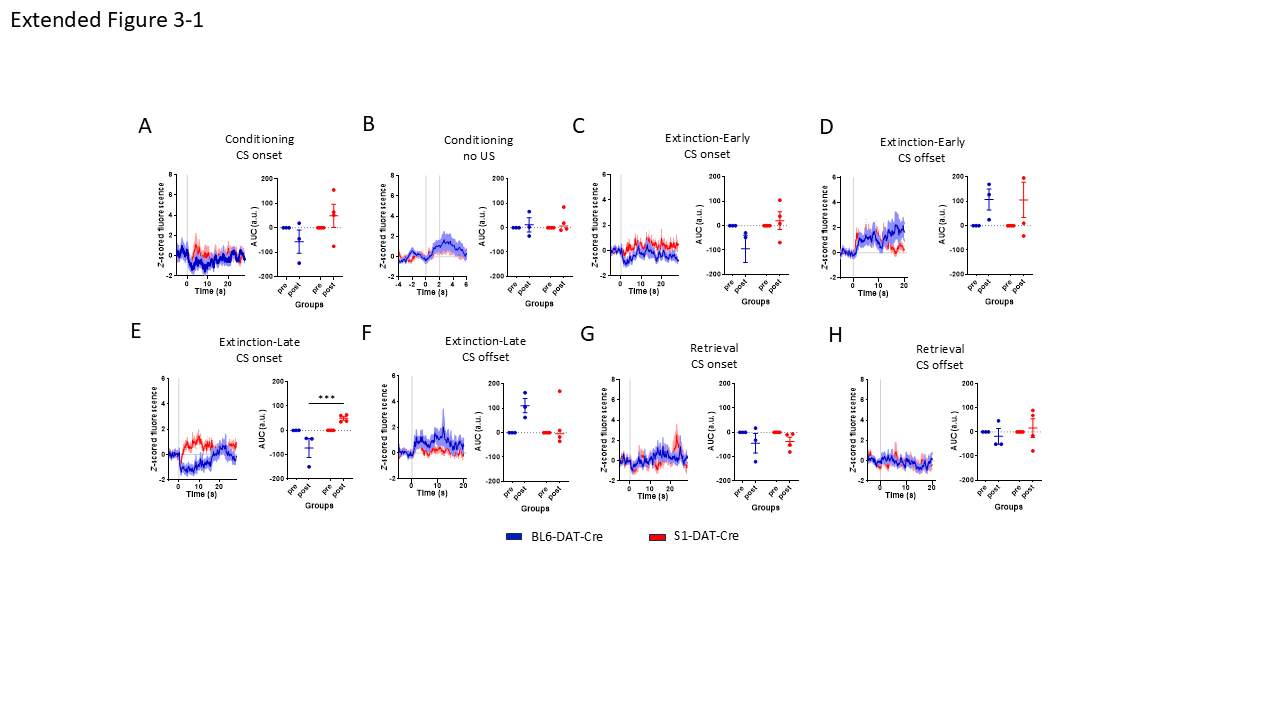

Supplement: Figure 3-1 — Fiber photometry Ca2+ measurements in the VTA during CS/no-US fear conditioning. All graphs show average Z-scores of changes in fluorescence (dF/F) for CS presentations baselined to 5 seconds before CS onset or CS offset and corresponding AUC values. A. There was no strain difference in fluorescence after CS onset during fear conditioning. B. Fluorescence at CS offset (2 seconds without US delivery) was similar between S1-DAT-Cre and BL6-DAT-Cre mice. C. During early EXT (First 10 CS trials), fluorescence at CS onset was not significantly different between strain. D. Fluorescent was not different between strains at US omission during early EXT. E. During late EXT (Last 10 CS trials), fluorescence at CS onset was not different between strains. F. During late EXT, fluorescence at US omission was similar across strains. G and H. Both strains no change in fluorescence at CS-onset and CS-offset during RET. S1-DAT-Cre GCaMP6 (red): n=4; BL6-DAT-Cre GCaMP6 (blue): n=3. Data are means ± SEM. Download Figure 3-1, TIF file. [file eneuro-12-ENEURO.0174-25.2025-s004.tif]

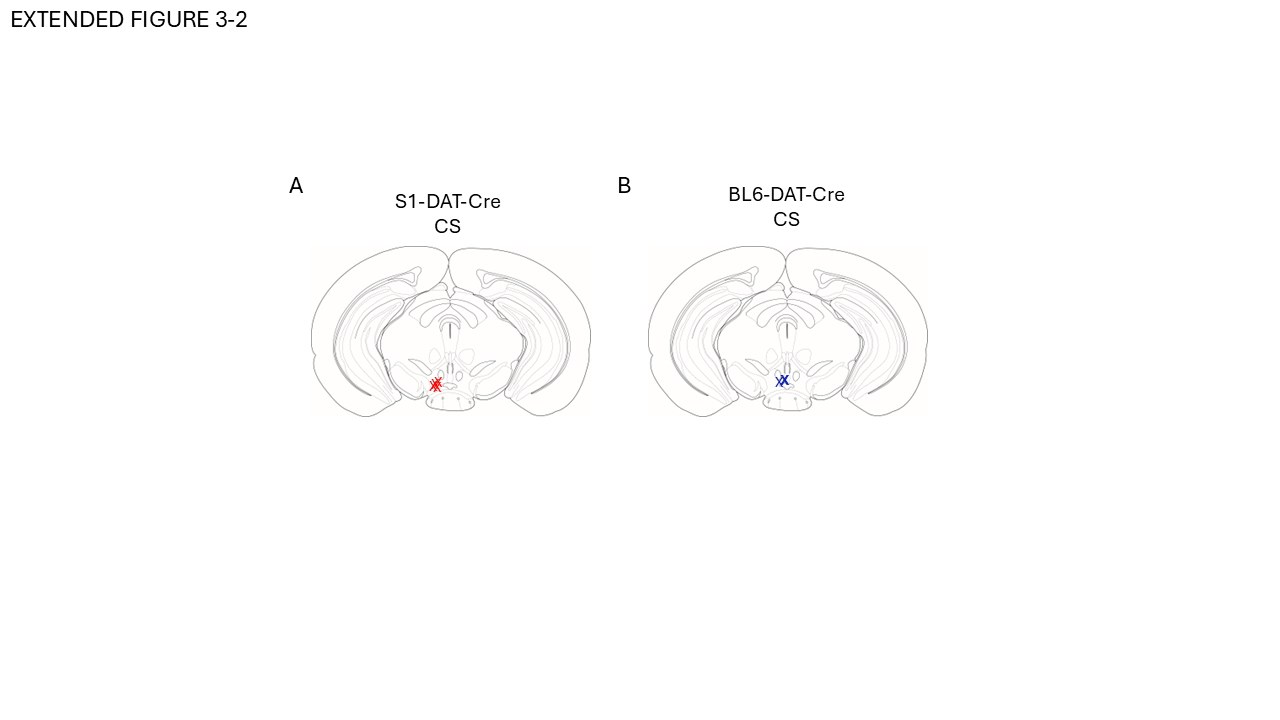

Supplement: Figure 3-2 — Fiber placement maps for fiber photometry experiments. A. Optical fiber placements for the fiber photometry experiments with S1-DAT-Cre mice (CS-only; n=4). B. Optical fiber placements for the fiber photometry experiments with BL6-DAT-Cre mice (CS-only; n=3). Download Figure 3-2, TIF file. [file eneuro-12-ENEURO.0174-25.2025-s005.tif]

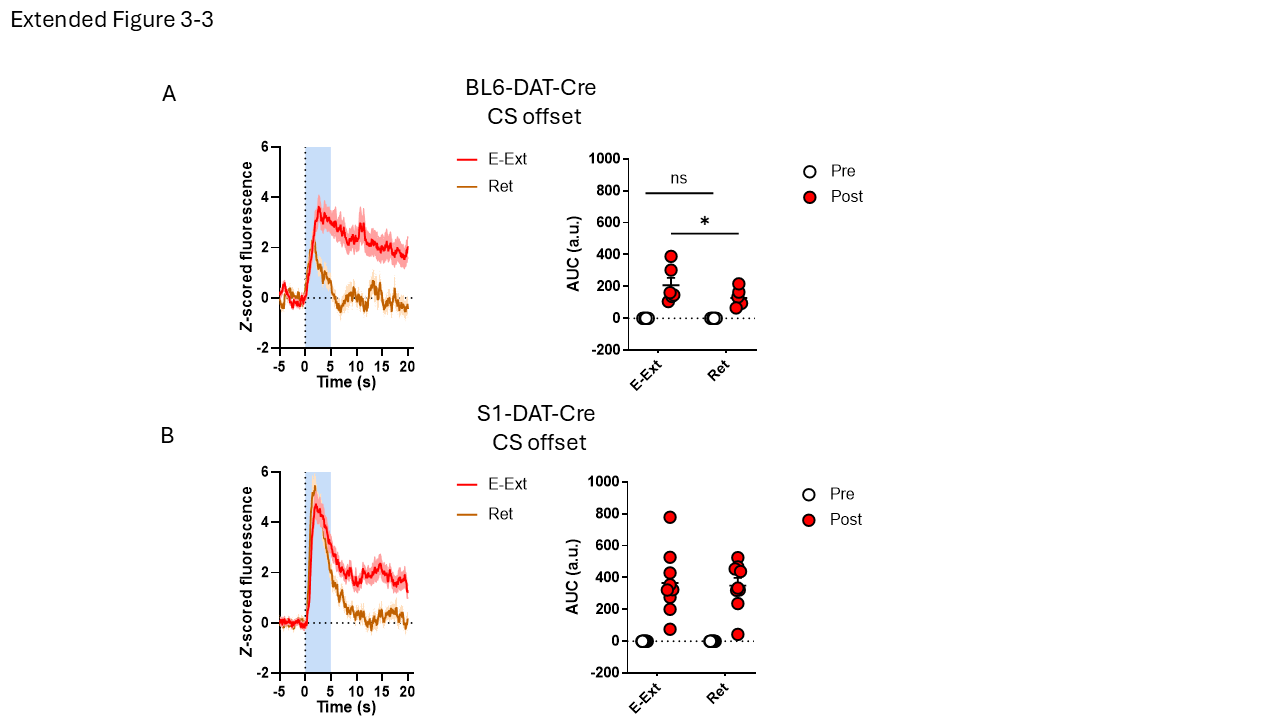

Supplement: Figure 3-3 — Fiber photometry Ca2+ measurements in the VTA during CS offset in Early Extinction and Retrieval. All graphs show average Z-scores of changes in fluorescence (dF/F) for CS presentations baselined to 5 seconds before CS offset and corresponding AUC values. A. There was a significant difference in fluorescence at CS offset in BL6-DAT-Cre mice (n=6) on RET compared to early EXT (First 10 CS trials). B. Fluorescence at CS offset remained the same in S1-DAT-Cre mice (n=9) on RET compared to early EXT. Data are means ± SEM. Download Figure 3-3, TIF file. [file eneuro-12-ENEURO.0174-25.2025-s006.tif]

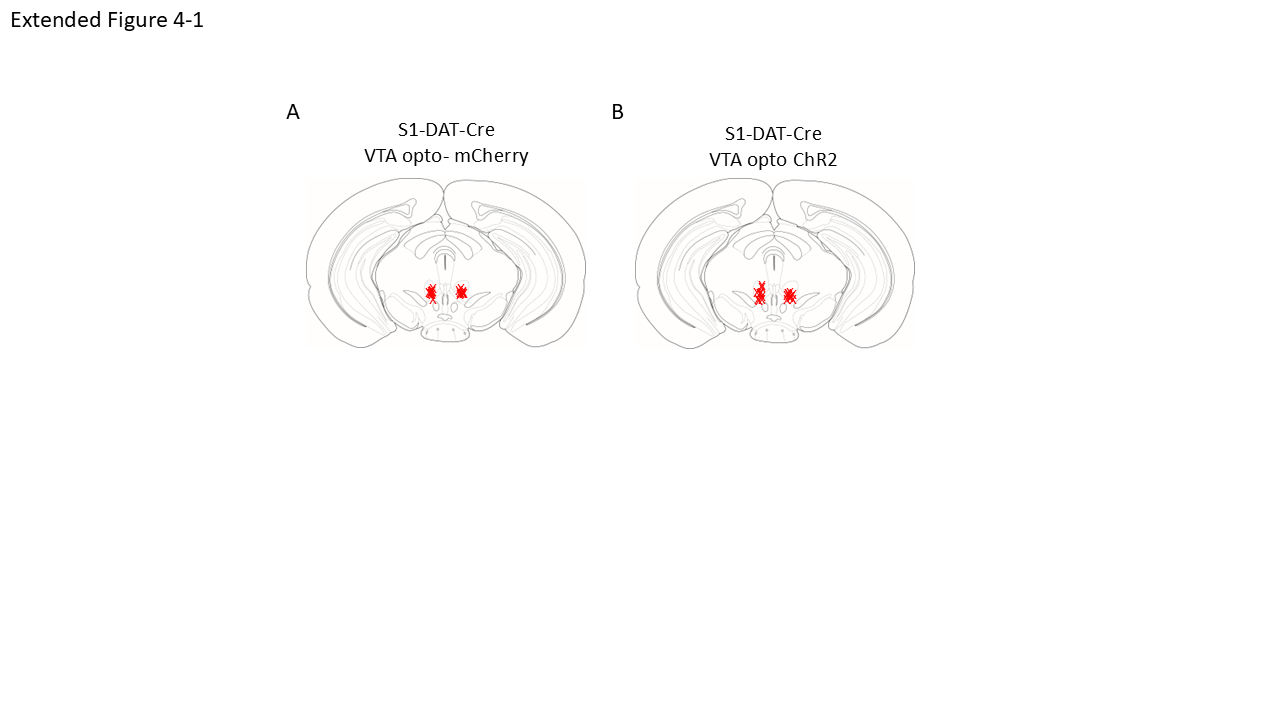

Supplement: Figure 4-1 — Optrode placement maps for optogenetic experiments (VTA). A. Optrode placements for the S1-DAT-Cre mCherry group shown in Figure 4 (n=10). B. Optrode placements for the S1-DAT-Cre ChR2 group shown in Figure 4 (n=12). Download Figure 4-1, TIF file. [file eneuro-12-ENEURO.0174-25.2025-s007.tif]

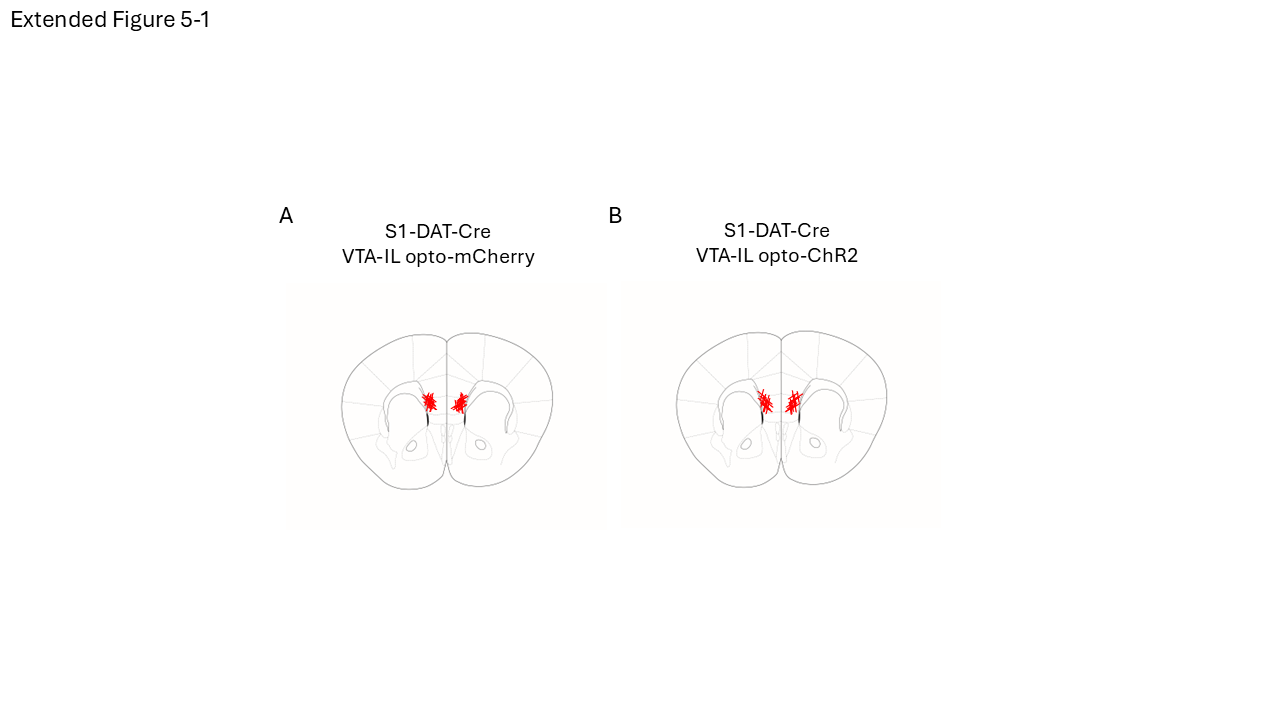

Supplement: Figure 5-1 — Optrode placement maps for optogenetic experiments (VTA-IL). A. Optrode placements for the S1-DAT-Cre mCherry group shown in Figure 5 (n=11). B. Optrode placements for the S1-DAT-Cre ChR2 group shown in Figure 5 (n=9). Download Figure 5-1, TIF file. [file eneuro-12-ENEURO.0174-25.2025-s008.tif]
